# Gymnosperm Resprouting—A Review

**DOI:** 10.3390/plants10122551

**Published:** 2021-11-23

**Authors:** Geoffrey E. Burrows

**Affiliations:** School of Agricultural and Wine Sciences, Charles Sturt University, Locked Bag 588, Wagga Wagga, NSW 2678, Australia; gburrows@csu.edu.au

**Keywords:** axillary meristems, buds, conifer, coppice, epicormic, layering, leaf axil, lignotubers, meristems, pine, sprouts, suckers, vegetative

## Abstract

Gymnosperms are generally regarded as poor resprouters, especially when compared to angiosperms and particularly following major disturbance. However, is it this clear-cut? This review investigates two main aspects of gymnosperm resprouting: (i) various papers have provided exceptions to the above generalization—how frequent are these exceptions and are there any taxonomic trends?; and (ii) assuming gymnosperms are poor resprouters are there any anatomical or physiological reasons why this is the case? Five of six non-coniferous gymnosperm genera and 24 of 80 conifer genera had at least one species with a well-developed resprouting capability. This was a wider range than would be expected from the usual observation ‘gymnosperms are poor resprouters’. All conifer families had at least three resprouting genera, except the monospecific Sciadopityaceae. Apart from the aboveground stem, buds were also recorded arising from more specialised structures (e.g., lignotubers, tubers, burls and underground stems). In some larger genera it appeared that only a relatively small proportion of species were resprouters and often only when young. The poor resprouting performance of mature plants may stem from a high proportion of apparently ‘blank’ leaf axils. Axillary meristems have been recorded in a wide range of conifer species, but they often did not form an apical dome, leaf primordia or vascular connections. Buds or meristems that did form often abscised at an early stage. While this review has confirmed that conifers do not resprout to the same degree as angiosperms, it was found that a wide diversity of gymnosperm genera can recover vegetatively after substantial disturbance. Further structural studies are needed, especially of: (i) apparently blank leaf axils and the initial development of axillary meristems; (ii) specialised regeneration structures; and (iii) why high variability can occur in the resprouting capacity within species of a single genus and within genera of the same family.

## 1. Introduction

The gymnosperms first appeared in the fossil record about 300 Mya, with the first angiosperms appearing about 135 Mya (based on fossil pollen) [1]. Conifers were widespread and dominant, especially during the Triassic and Jurassic periods. Extant gymnosperms number about 1000 species (conifers around 600 species and non-conifers, mainly cycads and gnetophytes, the other 400 species), compared to over 350,000 species of angiosperms. While conifers cover much of North America and Eurasia species diversity can be greater towards the equator, e.g., New Caledonia 43 spp. compared to all of Europe 41 spp. [1]. A major decline of gymnosperms and a rapid diversification of the angiosperms occurred during the Cretaceous [2]. Condamine et al. considered that direct competition with angiosperms resulted in increased extinction of gymnosperms, especially during the mid-Cretaceous. The increased competitiveness of angiosperms has been associated with numerous anatomical, morphological and physiological developments related to reproductive biology, water-conducting systems, secondary metabolisms and stress defence mechanisms [3].

Gymnosperms are generally regarded as poor resprouters, while most woody angiosperms are accomplished resprouters after substantial disturbance (e.g., storm damage, coppicing, herbivory and even after crown fire). Supplementary Table S1 provides quotes supporting this statement from numerous authors, over an extended period and from different disciplines (e.g., forestry and ecology). Additional supporting evidence is also available from the fields of arboriculture and horticulture (e.g., formative pruning). Most pruning books suggest that conifers should only be pruned to where green leaves are present and not to prune into brown old wood for most species [4,5]. From an ecological perspective Stahl et al. (2013) [6] assessed 23 functional traits of 305 North American woody species (103 gymnosperm species, 202 angiosperm species) to determine their ecological strategies. Three leaf traits, conduit type (tracheids vs. vessels) and resprouting capacity were mainly responsible for the separation of gymnosperms and angiosperms. Bond (1989) [7], in his slow seedling hypothesis, also notes gymnosperms have a more rigid architecture and are slower and less likely to reiterate branches into canopy gaps.

In contrast to most of the general statements in Supplementary Table S1, various authors have prepared listings of gymnosperms that could resprout or had some form of vegetative regeneration (e.g., [8,9,10,11]). Farjon [12] provides extensive conifer resprouting observations that are included within the individual species descriptions in ‘Conifers of the World’. Burns and Honkala [13] provide detailed information on vegetative reproduction for 65 conifer species of North America (see also the USDA Fire Effects Information System website https://www.feis-crs.org/feis/ (accessed on 1 September 2021) for further information regarding the resprouting of gymnosperms of the USA). The first comprehensive compilation of conifer vegetative reproduction was that of Lamant [14], although this assessment indicated that additional information was needed for 26 genera. Chris Earle (see the website ‘The Gymnosperm Database’ https://www.conifers.org/ (accessed on 1 July 2021), which includes the page ‘vegetative reproduction in conifers and *Ginkgo*’ https://www.conifers.org/topics/vegetative.php) (accessed on 1 July 2021) organised Lamant’s information into a list and updated some aspects. He et al. [15] (their Supplementary Table S3) compiled a list of gymnosperms (about 40 genera and 95 taxa) with resprouting capacity.

Various authors have provided data that quantify that angiosperms are better resprouters than gymnosperms. Del Tredici [11] noted that of 68 temperate tree species (13 conifer species, 55 angiosperm species) of NE North America, 78% of the angiosperms could resprout as adults, while none of the conifers could. Vesk and Westoby [16], based on a literature search of mainly Australian and North American species, recorded that of nine gymnosperm species, six were non-resprouters, two were intermediate and one was a resprouter. Gymnosperms had the highest percentage of non-resprouters of 19 high level taxonomic groupings. Paula et al. [17] assessed 13 Mediterranean conifers for their capacity to resprout after fire. Ten species were recorded as non-resprouters (mainly species of *Pinus*), one was a resprouter (*Tetraclinis*) and two had variable resprouting reports. Pausas et al. [18] investigated belowground bud banks in ecosystems with severe recurrent disturbance (fire). In the supporting information they provide data on 21 genera and 37 species of conifers and three species of non-coniferous gymnosperms.

He et al. [15], in an investigation of serotiny, presented evidence that fire had influenced plant evolution from 350 million years ago. They also briefly mentioned other fire-related traits such as shedding of dead branches, bark thickness and resprouting. They considered that resprouting was relatively common in extant conifers. They indicated that there were at least 41 conifer genera and at least 94 coniferous taxa that have a resprouting capability after disturbance. He et al. [15] and its supporting Table S3 are currently the most detailed information source on resprouting in the gymnosperms. In terms of indicating why the present survey was carried out it should be noted that the information in He et al.’s Table S3 needs extending as: (i) the table is listed as covering gymnosperms but includes only one of over 400 non-coniferous gymnosperm species, (ii) apart from Lamant [14] only a further 11 references are used and (iii) ‘resprouting modes’ for the taxa are given as 80, 17 and 3% epicormic, layering and suckering, respectively. The disturbance would need to be quite slight to have such a high percentage of epicormic resprouting.

On the basis of the information in Supplementary Table S1, it would appear that gymnosperms are relatively poor resprouters, but in several of these papers (e.g., [19,20]) a few exceptions are provided. When these exceptions were compiled, there were enough to suggest that gymnosperms were better at resprouting than is usually stated. Thus, this review has the following aims:

(i) To conduct an extensive literature review of resprouting of gymnosperms, including a more in-depth analysis of some of the better resprouting genera (e.g., *Araucaria, Pinus*). While focussed on resprouting after substantial disturbance (e.g., coppicing, severe fire (e.g., 100% leaf scorch of the plant), information on less severe damage (e.g., formative pruning, frost, insect attack) and other forms of vegetative reproduction (e.g., layering) was also recorded. This was conducted as any resprouting is indicative of a long-lived bud reserve, which is an important but little studied aspect of gymnosperm resprouting.
(ii) To conduct a taxonomic assessment of the literature review at a species, genus and family level.
(iii) To assess gymnosperm resprouting in the fossil record.
(iv) To assess the reasons why many gymnosperms are poor resprouters. Resprouting requires buds, as well as protection and resources for those buds (Clarke et al., 2013). Does studying the ontogeny of axillary buds and meristems help understand gymnosperm resprouting?

## 2. Taxonomic Assessment

A systematic review of the gymnosperm resprouting literature was undertaken. All families and genera were searched against terms such as ‘resprout*’, ‘coppice’, ‘epicormic’, ‘vegetative’, ‘asexual’, ‘layering’, ‘suckering’, ‘root shoot’ and ‘fire’ in Google, Google Scholar and Web of Science. The main previous studies on this topic used six [14], 15 (Earle 2020 website) and 12 [15] references. This study is based on around 220 references (Supplementary Tables S2 and S3).

**Figure 1:**
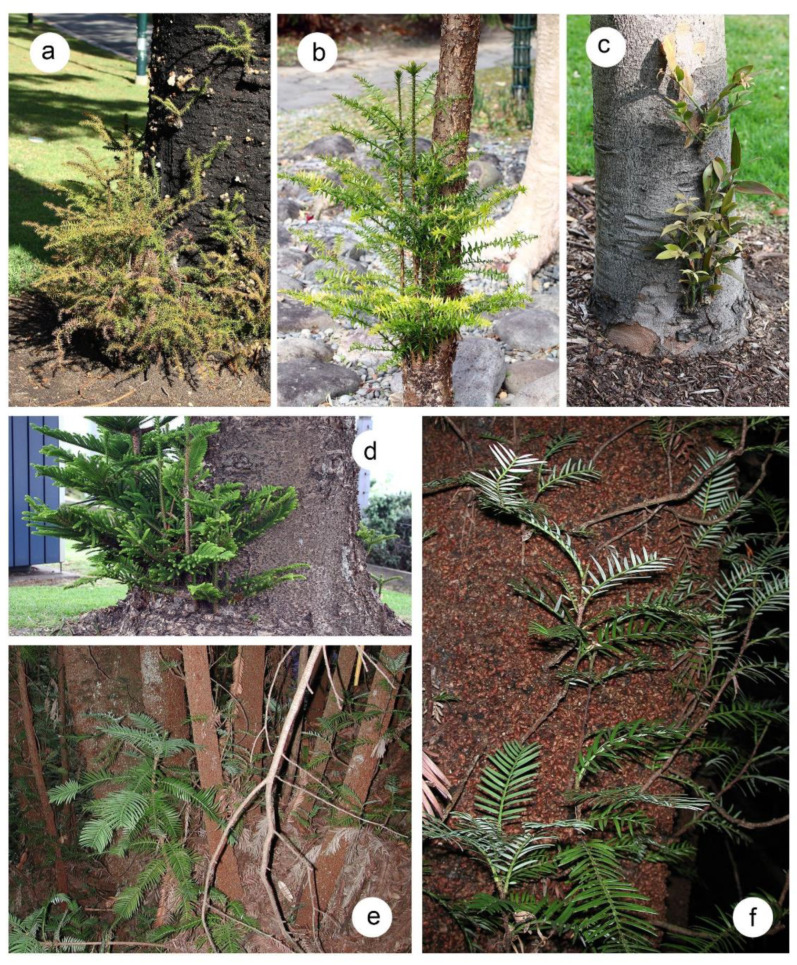
Epicormic and coppice shoots for various species in the Araucariaceae. (**a**) *Araucaria cunninghamii*, (**b**) *Araucaria bidwillii*, (**c**) *Agathis robusta*, (**d**) *Araucaria heterophylla*, (**e**,**f**) *Wollemia nobilis*. In (**a**–**d**) the shoots had formed on relatively intact specimen trees that may have been subject to damage from landscape maintenance. While different to resprouting after substantial damage it does show that these conifer species retain a resprouting capacity in mature trees. (**e**,**f**) *Wollemia nobilis* in the Wollemi National Park. (**e**) clump of coppice shoots from what is possibly a single individual. (**f**) large diameter trunk with numerous epicormic shoots growing from the distinctive bubble bark.

**Figure 2:**
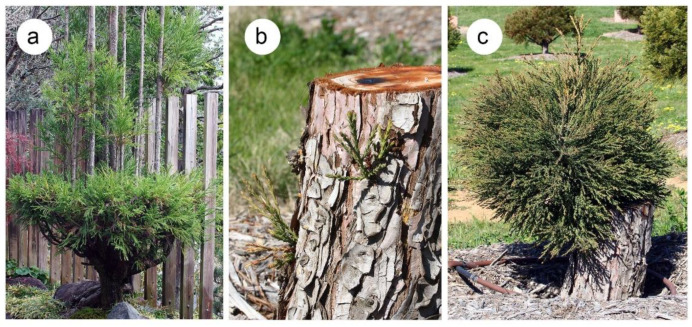
Epicormic resprouting in two species of the Cupressaceae. (**a**) *Cryptomeria japonica*. This species is used for bonsai and topiary and this example in a Japanese botanical garden had been pruned to form several leaders of equal dominance. (**b**,**c**) *Sequoiadendron giganteum*. This species is known to resprout from the stumps of younger trees, as is shown here for trees in an arboretum.

Four of the six (mainly monogeneric) families of non-conifer gymnosperms each had a resprouting genus (Table 1, Supplementary Table S2). Within the conifers, 23 of the 70 genera were assessed to have at least one species that was capable of resprouting after substantial disturbance (Figure 1, Figure 2). Other genera such as *Athrotaxis*, *Diselma*, *Fitzroya*, *Pilgerodendron*, *Lagarostrobos*, *Microcachrys* and *Pherosphaera* can vegetatively reproduce via root suckers and/or buried stems as a normal part of plant development, in some cases forming clonal patches. This is not considered in this review as ‘strongly resprouting’ but perhaps could be. At least three resprouting genera were identified in all families except for the monospecific Sciadopityaceae. In the Araucariaceae, all three genera had resprouter species (Figure 1), while only four of the 19 Podocarpaceae genera were classified as accomplished resprouters. At a generic level, it appears that all 19 species of *Araucaria* could resprout after branch or leader damage (i.e., coppicing but not necessarily fire), while in some of the other larger genera (e.g., *Juniperus* (75 spp.)*, Pinus* (111 spp.) and *Podocarpus* (110 spp.)) there was a mix of resprouters and non-resprouters, with the latter in greater abundance. This dichotomy of resprouting responses in closely related species can be found in other genera, even ones that are excellent resprouters. For example, in the eucalypts several pairs of taxa are morphologically very similar, except for presence/absence of a lignotuber [21]. One taxon from each pair has gained or lost the ability to form lignotubers. In summary, this review (Supplementary Table S2) indicates that gymnosperms are indeed generally poor resprouters; however, there are more exceptions to this generalisation than are generally known. These exceptions are spread across a wide range of genera and families and some of the exceptions are excellent resprouters, with similar capabilities to angiosperms.

**Figure 3:**
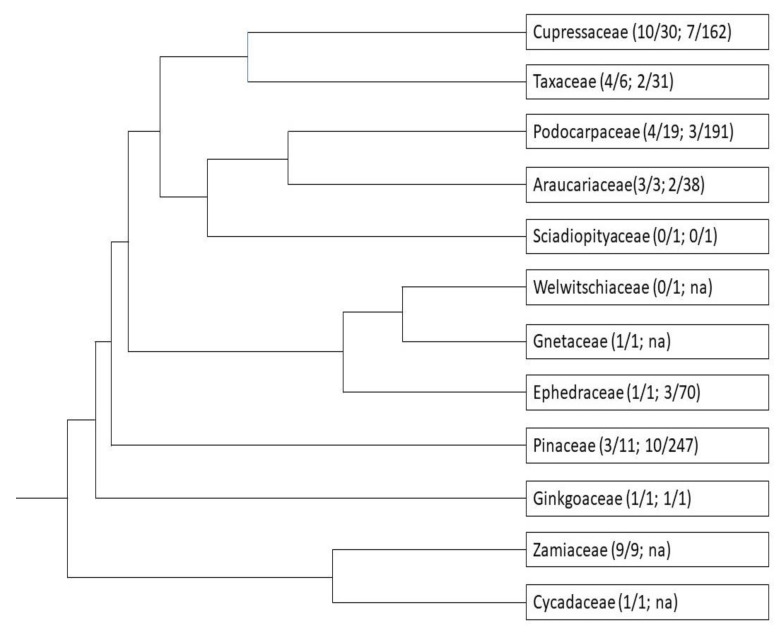
Phylogeny of extant conifer families (adapted from Figure 5 of Lu et al. [23]). Numbers in brackets represent number of resprouting genera/number of genera in the family; number of species resprouting after 100% leaf scorch/total number of species in the family.

Various fire-related traits of gymnosperms have been mapped on to phylogenetic trees. He et al. [22] found that fire-protective thick bark developed in *Pinus* approximately 126 Ma and at around 89 Ma even thicker bark combined with branch shedding or serotiny without branch shedding appeared. The grass stage (see Section 7.1.) was rare and clearly derived with lineages traceable to <40 Ma. Sexual reproduction characteristics such as seed cones with woody rachis, seed cones with woody scales and winged seeds have also been mapped on to phylogenetic trees for gymnosperms [15]. Figure 3 shows a phylogeny of extant gymnosperm families. It shows that strongly resprouting species/genera are widespread across the 12 families, except in the monospecific Sciadopityaceae. Some traits related to recovery from major disturbances have been mapped onto phylogenetic trees (see above). Usually, these traits are binary in nature. This is not the case for resprouting capacity as: (i) species and genera quantified in Figure 3 have varied resprouting structures and types (e.g., epicormic, basal, lignotubers, root shoots, rhizomes, tubers), (ii) capacity differs widely between seedlings, saplings and mature trees, (iii) different disturbances (e.g., fire (of varying intensity), coppicing, pruning, herbivory) produce different resprouting responses and iv) several genera (e.g., *Juniperus, Pinus, Podocarpus*) have a mix of poor and accomplished resprouters. Probably the strongest phylogenetic trend is the apparently unique axillary meristems and bark patches in the Araucariaceae (see Section 4.4, Section 5 and Section 6) which, according to the divergence times of Lu et al. [23], originated at least 190 Ma.

## 3. Fossil Evidence of Resprouting

Both the fossil record and molecular phylogenetic evidence indicate that the conifers originated during the middle Carboniferous (340–310 Mya) and rapidly diversified during the Carboniferous and Permian [24]. This was a time when fire was widespread [15]. Fire tolerance is a general trait in conifers [24], with extant conifer families exhibiting various fire-adapted traits, e.g., serotinous cones, thick bark and lower branch shedding. As previously indicated, He et al. [15] identified 41 gymnosperm genera with resprouting or vegetative reproduction capacity and it has been concluded that resprouting had an early origin in conifer phylogeny [24]. While molecular phylogenetic analysis has been used in assessing the evolution of epicormic resprouting in some groups of angiosperms (e.g., Myrtaceae [25]), is there fossil evidence for resprouting in conifers?

Several reports of vegetative reproduction in fossil gymnosperms have been published, including epicormic resprouting and production of root shoots. Epicormic resprouting has been recorded in *Woodworthia* [26,27], Glossopterids [28], *Araucaria mirabilis* [29], *Cuyoxylon* [30], a new tree taxon [31] and an unspecified conifer genus [32]. Root suckering has been recorded in *Notophyllum krauselii* [33,34] and *Austrocupressinoxylon barcinense* [35].

The above studies of epicormic resprouting were mainly based on decorticated fossil stems of a wide range of diameters (3–76 cm) and from various geological periods, e.g., Palaeozoic, Triassic and Jurassic. Evidence for the previous existence of epicormic buds or shoots on these stems was based on: (i) patterns on the surface of the cylinders (e.g., cylindrical/circular projections or depressions) [26,28,30] and/or (ii) the presence of radial vascular traces within the xylem [26,27,28,30]. Comparison was made between these structures and epicormic structures in extant gymnosperms. Falaschi et al. [29] indicated the occurrence of both total (new orthotropic leaders) and partial (epicormic shoots) reiteration in *Araucaria mirabilis*. Most studies considered the buds to be preventitious but it was also suggested they may have been of adventitious origin [28,29,30].

Several of these studies indicated that the epicormic shoots would have had similar functions (normal part of crown maintenance (i.e., non-substantial disturbance) as well as recovery after traumatic events) to that described for extant trees, both gymnosperms and angiosperms. In terms of traumatic injury, Falaschi et al. [29] noted that the fossil trunks they studied were growing in volcanic palesols and the reiterations were for repairing damage, probably volcanic in origin. Thus, gymnosperms have a long history of clonal growth through root shoots as well as responding to damage through epicormic shoots. He et al. [15] noted that the ability of a wide range of modern conifers to resprout after disturbance implies that this capability must have originated early in conifer phylogeny, which aligns with selective pressure from recurrent fires in the Paleozoic. The above fossil records of resprouting add weight to He et al.’s hypothesis.

## 4. Axillary Buds and Meristems

### 4.1. Introduction

Without buds or their precursor meristems, resprouting will not occur [36]. As eloquently stated by Stone [37] (p. 366), “The presence of buds can be readily determined in advance: No buds; no sprouts!”. The presence of buds does not ensure that resprouting will occur, but it is a vital preliminary step. As most coppice and epicormic shoots arise from preventitious buds it is important to assess the initial formation of axillary buds and/or meristems. While adventitious buds can form on gymnosperm roots (e.g., [38]) and possibly on trunks (see [39,40] for angiosperm examples) these de novo buds are responsible for a small proportion of shoots [41], except in the cycads where it is considered that axillary buds do not form and adventitious buds can develop from damaged regions and leaf bases (Supplementary Table S2) [42]. Farjon [12] frequently mentions adventitious buds and adventitious foliage branches, especially in *Araucaria* but also in *Dacrycarpus*, *Pinus*, *Taxodium* and *Tetraclinis*. It is likely (see the following sections) that these buds and branches are preventitious in origin. Thus, an assessment of axillary meristem initiation and subsequent ontogeny is critical to understanding the different resprouting capabilities of gymnosperms versus angiosperms and between gymnosperm species of the same genus and genera in the same family. The angiosperms have resprouting potential in almost all leaf axils, usually in the form of fully formed buds (apical dome with overarching leaf primordia) with vascular connections from the buds to the central stele. Multiple buds in a single axil (e.g., accessory buds) form in some species. There can be a wide variation in anatomy between species, even in the same family (e.g., [43]), but only a few reports of ‘blank’ or ‘empty’ axils (i.e., without macroscopically visible buds) have been made. This review will first assess bud forming structures in gymnosperm leaf axils, then the structures they can develop into as plants age or increase in diameter, e.g., epicormic buds, basal buds and lignotubers.

### 4.2. Non-Coniferous Gymnosperms

The cycads (Cycadaceae 92 spp., Zamiaceae 216 spp.) are generally considered to not form axillary buds, with adventitious buds able to form in a wide range of organs and tissues, with and without damage to a plant [42]. *Ginkgo biloba* (Ginkgoaceae 1 sp.) produces long shoots with well-developed internodes and axillary buds, as well as short shoots with very short internodes and no axillary buds [44]. In addition, lignotubers with numerous buds develop from buds in the axils of the cotyledons [44]. *Ephedra* (Ephedraceae 65–70 spp.) has early development of conspicuous axillary buds, although in *Ephedra foliata* in nodes with four leaves only one of the leaves will have an associated axillary bud [45,46]. In *Ephedra pedunculata* buds can form in the axils of the cotyledons and more than one bud may form in an axil [47]. In the leaf axils of *Gnetum gnemon* (tree) and *Gnetum africanum* (vine) (Gnetaceae 41 spp.), a normal axillary bud is present (upper position) along with a collateral supplementary bud (lower position) that functions as a suppressed and inconspicuous bud reserve [48,49,50]. Some species also produce ‘root tubers’ or ‘underground tubers’. As tubers can be the swollen part of either a stem or root, the buds they produce might be either preventitious or adventitious. In *Welwitschia mirabilis* (Welwitschiaceae 1 sp.) the seedling shoot apex produces three pairs of leaves before it become meristematically inactive [51,52]. The second pair of leaves is capable of growing indefinitely through a basal leaf meristem. The only axillary activity is related to the production of reproductive structures.

These non-coniferous gymnosperms have a wide variation in axillary structures (apparently blank or empty leaf axils to axils with collateral buds, as well as buds on tubers, rhizomes and lignotubers) and generally these morphological and anatomical observations correlated well with their recorded resprouting responses (Supplementary Tables S1 and S2). It should be noted that bud information for *Ephedra* and *Gnetum* is based on data for only a small percentage of the 40 plus species in each genus.

### 4.3. Conifers—General

As conifers are generally considered to have a high percentage of apparently blank or empty leaf axils [53,54,55] at least some of their poor resprouting capabilities could relate to this lack of meristematic potential. Holthusen [56] examined leaf axil structure in 28 conifer species in four families. With the exceptions of, for example, *Taxus baccata, Abies concolor* and *Pinus* spp., the leaf axils examined were recorded as being devoid of buds or meristems. However, subsequent investigations have re-examined some of the species studied by Holthusen and found axillary meristems (e.g., *Sequoiadendron giganteum* (syn. *Sequoia gigantea*) [55] and *Araucaria bidwillii, Araucaria cunninghamii* and *Araucaria heterophylla* (syn. *Araucaria excelsa*) [53,54]). The meristems in these species are very small (see below) and thus might have been overlooked.

Fink [55] examined the leaf axils of five species from the Cupressaceae (species of *Cryptomeria, Sequoia, Sequoiadendron, Thuja, Thujopsis*) and *Taxus baccata* (Taxaceae). In *Taxus baccata* persisting detached meristems were located in the apparently empty needle axils. After two to four years these meristems gradually developed into small axillary buds. In *Sequoia sempervirens* persisting detached meristems formed singly in a leaf axil and in an accessory position (located between the regular axillary shoots and the subtending leaf). The other four species developed minute meristems in the leaf axils but they were abscised after one to several years when periderm formation began. These meristems may allow for resprouting after damage to young shoots. Interestingly *Taxus baccata* and *Sequoia sempervirens* are recorded as strong resprouters, while species of *Cryptomeria* and *Thujopsis* are generally considered poor epicormic resprouters (Supplementary Table S2). If these persisting detached meristems are widespread in the Cupressaceae and perhaps other conifer families, but only relatively short lived, this might indicate why many conifers can be hedged but the general pruning advice is not to cut back into wood where the leaves have fallen [4,5].

*Taxodium distichum* forms exogenous and ‘pseudo-endogenous’ axillary buds [57]. The pseudo-endogenous buds form in many, but not all, axils of the proximal leaves of expanding permanent shoots. Their development is delayed by two to three months compared to the exogenous buds, perhaps showing that the axillary cells have specific development or meristematic capabilities. This species is a prolific stump resprouter, from saplings to very old trees [13].

### 4.4. Araucariaceae

Mature trees of many species of the Araucariaceae can resprout (Supplementary Table S2), which has been reported after coppicing in forest operations and also after fire for *Araucaria araucana* [58] and *Wollemia nobilis* [59]. Several anatomical investigations of their axillary buds and meristems have been made. Holthusen [56] recorded that neither buds nor meristems were present in the leaf axils of three species of *Araucaria* and one species of *Agathis*. In contrast, for *Araucaria angustifolia*, Fink [39] recorded that small groups of cells of meristematic appearance were present in the axils of all leaves except where branch buds had formed. Fink recorded that cell division in the axils gave rise to small protrusions. It was considered that the outer cells of the protrusions soon vacuolated and only at the base did the cells retain their meristematic appearance. The meristems were considered to be endogenous in origin. Iritani et al. [60] also recorded the presence of axillary meristems in *Araucaria angustifolia*.

**Figure 4:**
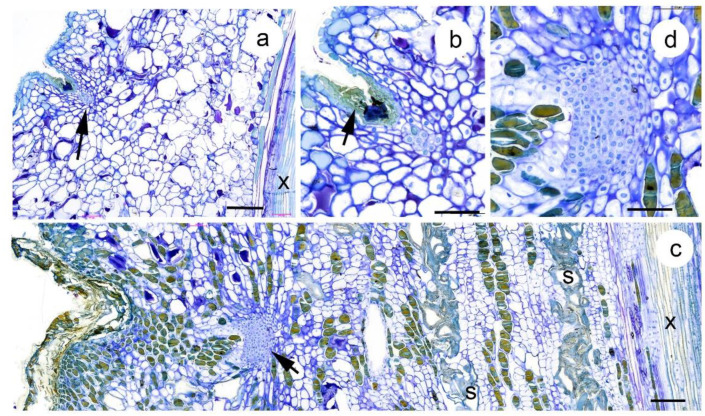
Radial longitudinal sections of the leaf axils of *Wollemia nobilis*. (**a**) Leaf axil from a plagiotropic first order branch of juvenile morphology. Note the location of the axillary meristem (arrowed) and the xylem of the central vascular cylinder (x). Scale 200 μm. (**b**) Detail of a) showing the small axillary meristem (note the large nuclei and dense cytoplasm) and the bark patch (arrow), the activity of which resulted in the axillary meristem being located below stem level. Scale 100 μm. (**c**) Leaf axil from the orthotropic leader of a mature tree. Note the xylem (x) of the central vascular cylinder, the sclereid (s) bands in the cortex and the relatively deeply buried axillary meristem (arrowed). Scale 200 μm. (**d**) detail of (**c**) showing the axillary meristem has a distinct outer layer, while it gradually merges into the cortex to the rear. Note the absence of leaf primordia or vascular connections. Scale 100 μm.

Burrows [53,54] described the formation of detached axillary meristems, similar to those of *Araucaria angustifolia* [39], in the leaf axils of six species of *Agathis* and 14 species of *Araucaria*. The cells in the axils were of meristematic appearance but there were no additional divisions in the meristems (thus no apical dome, no leaf primordia and no vascular connections). In contrast to Fink, Burrows [54] found that the axillary meristems of *Araucaria cunninghamii* were exogenous, but were subsequently buried beneath the stem surface by the activity of very localised phellogens adjacent to the meristems, resulting in the formation of small ‘bark patches’. There was strong but indirect evidence of a similar ontogeny in several of the other investigated araucarian species [53]. Iritani et al. [60] illustrated that bark patch formation also occurred in *Araucaria angustifolia*, indicating a similar ontogeny to that of *Araucaria cunninghamii*. The phellogen of the bark patch subsequently provided a path for the later formed extensive phellogen to differentiate to the outside of the meristems and consequently they were not abscised. In addition, *Wollemia nobilis* was also found to have the same type of axillary meristems as *Agathis* and *Araucaria* [61] (Figure 4). *Wollemia nobilis* can produce epicormic shoots on intact trees (Figure 1f). Some of the axillary meristems can form small buds and vascular tissue within the bark [62], which is probably a preliminary step in producing these shoots. These araucarian axillary meristems were very small (usually less than 140 μm in radial, vertical and tangential dimensions) in the leaders of seedling to adult plants of *Araucaria cunninghamii* and were progressively smaller in the leaf axils of the first, second and third order branches [54]. Likewise the main stem axillary meristems of *Wollemia nobilis* were larger (130–210 μm diameter) than those in the first order branches (45–75 μm diameter) [61] (Figure 4). For *Larix occidentalis* Owens and Molder [63] suggested that the absence of buds in the leaf axils may be related to a lack of sufficient internodal space. Compared to buds, araucarian detached meristems require no additional volume and can thus occur where numerous small needles are clustered together.

For *Wollemia nobilis* Tomlinson and Huggett [64] suggested that the axillary buds that formed after damage to first order branches arose from the dedifferentiation of cells in the leaf axils. They suggested that persistent meristems in these axils were hypothetical. They used 60–90 μm thick sections, which was not ideal when searching for structures only 45–75 μm in diameter [65]. In addition, they overlooked information that indicated persistent axillary meristems had already been described and illustrated in leaf axils of first order branches of wollemi pine [61,65].

The araucarian axillary meristems are very consistent in structure across the family and different to that described for other conifers. While approaching the angiospermous condition of having long-lived regenerative potential in every axil, they differ in that the axillary meristems do not form an apical dome or leaf primordia and have no vascular connections until they receive hormonal signals (e.g., after damage) to develop into buds. This comparatively independent existence within the stem’s cortex leads to other unusual developmental consequences as the stems increase in diameter (see subsequent section).

### 4.5. Pinus

*Pinus* seedlings initially produce numerous narrow elongated primary leaves before the shoot apical meristem starts to produce the characteristic dwarf shoots in the axils of scale leaves [66,67]. Lester [66], who investigated seedlings of nine *Pinus* species over a period of 120 d, described three zones of differing axillary activity. For several of the species, there was a proximal zone where many of the primary leaf axils were blank (20–40% of axils had lateral branches of indeterminate growth, i.e., long shoots), then a middle zone with few (0–25%) axillary meristems, then a distal zone with 80–100% dwarf or short shoots. Stone and Stone [68] considered that each of the lower primary needles of *Pinus* seedlings contained a potential bud-forming meristem but in some species they remain blank, while in others an abundance of axillary shoots formed.

*Pinus* dwarf shoots (also known as brachyblasts or needle fascicles) are normally regarded as a determinate short shoot [69] but can have an interfoliar bud [70]. This is a persistent terminal dwarf shoot apical meristem [69] that can proliferate into long shoots, either after shoot damage or, more rarely, as a normal part of development [71,72]. In 23 *Pinus* species Doak [71] found that ‘growing points’ were present in at least some of the dwarf shoots. In contrast, in *Pinus monophylla* after the single needle in the dwarf shoot is initiated the apex becomes inactive and decreases in size before becoming “obliterated” [73]. In *Pinus lambertiana* most of the outer cell layers of the residual apices of the dwarf shoots undergo dessication [70]. In *Pinus longaeva* the distal dwarf shoots had interfoliar buds that could develop into long shoots, while proximal dwarf shoots lacked protective scales and often had a suberised apex that could not proliferate [69,72]. Given that the dwarf shoot retention times for many low elevation pines in North America are around two to four years [74], even if an individual dwarf shoot possesses an interfoliar bud, this is not a long-lived resprouting resource. Little and Somes [75] noted that for *Pinus echinata* and *Pinus rigida* the needle fascicle buds were relatively unimportant in recovery from injury.

Del Tredici [11] made the broad generalisation that conifers with two cotyledons have cotyledonary buds and collars that sprout, while conifers with three or more cotyledons lack cotyledonary buds and functional collars, with some *Pinus* species (see following section) as the main exception. Butts and Buchholz [76] provide cotyledon numbers for almost 200 species of conifers.

### 4.6. Conifers—Various

The preceding morphological and anatomical studies examined the axillary meristems of multiple species in the Araucariaceae and *Pinus*. The next section brings together information about axillary meristems of single species from a wide range of genera, mainly examining why relatively few buds form and why buds/meristems are not long-lived.

In saplings of *Abies nordmanniana* meristematic activity was visualised by ubiquitin immunohistochemical localisation. The staining indicated that the initial stages of axillary bud initiation began in the axils of all needles, but the signal transduction pathway did not proceed, meaning that buds were not formed except in a few axils [77]. In the same species, Briand et al. [78] noted that leaves appeared to have ‘empty’ axils but in certain axis orders buds could develop. They stated that the reiterated shoots were not adventitious and presumably originated from detached meristems.

In *Thuja plicata* seedlings, structures variously described as ‘meristematic zones’, ‘undifferentiated clumps’ and ‘rudimentary bud primordia’ were present in the axils of all leaves and were not linked to the stem’s vascular tissues [79]. They could, under certain conditions, give rise to axillary shoots.

While not specifically mentioning axillary meristems, Jansson and Bornman [80] noted that in needle explants of *Picea abies*, the area where the axillary buds would have formed, had there been any, had a high capacity for regeneration. They described a “peg-like cushion of axillary, meristematic tissue” (p. 191) proximal to the needle abscission zone that was capable of regeneration. Cannell et al. [81] considered that species of *Picea* and *Abies* do not produce true axillary buds. The buds formed by dedifferentiating areas of cortical tissues in a few needle axils of elongating preformed shoots “long after the needles were initiated” (p. 195).

As Fink [82] (p. 376) noted, “even in the absence of any buds or meristems, the axillary positions are predisposed for the neoformation of buds from already differentiated tissue by anaplasia”.

In *Cephalotaxus drupacea* there are empty leaf axils where the tissues of the axil differentiate as epidermis and cortical parenchyma, with no evidence of a bud initial [83]. If the terminal bud of a shoot is damaged, cells of the empty leaf axil can dedifferentiate and form a replacement meristem.

Harrison and Owens [84] described two types of bud damage in *Picea engelmannii*. Some buds were damaged by insects, while others degenerated or aborted. Sixty-six percent of axillary buds that developed on vegetative shoots became latent or there was degeneration or abortion of the bud. The 66% abortion estimate was considered conservative, as many buds may have aborted before they were large enough to be counted. This has also been noted in other species, e.g., *Tsuga mertensiana* [85].

*Cunninghamia lanceolata* is a prolific stump resprouter, especially in trees less than 30 years old [86]. Yanming and Jingzhong [87] described the presence of supressed axillary buds and persisting detached meristems, along with the absence of adventitious buds.

In *Thuja occidentalis* unbranched shoots from in vitro cultures were examined for bud histogenesis [88]. In the absence of supplied cytokinin few axillary buds developed. However, in most axils detached axillary meristems were present. In the absence of cytokinin the cells in the meristems underwent very few divisions and did not form an apical dome, leaf primordia or vascular connections.

Some studies have indicated that cytokinin sprays on conifers growing in the field can increase the number of visible axillary buds. Little [89] indicated that a 6-benzylaminopurine spray on five to six year old plants of *Abies balsamea* increased meristematic activity within needle axils, resulting in an increased number of lateral buds. In some shoots a bud was formed in almost every axil showing that the normal production of lateral buds was much less than the potential production. In a similar manner, Fink [82] indicated that after cytokinin treatment buds could be induced in all needle axils in shoots of *Abies alba*.

Combined, these observations, mainly for species in the Cupressaceae and Pinaceae, indicate that conifer leaf axils might not be as blank or empty, at least in recently initiated shoots, as they might appear to be. Cells in the leaf axils, while not forming buds, can maintain a meristematic potential and if they lose meristematic appearance, they may be preferentially able to dedifferentiate into bud forming structures. Nonetheless, it appears that development of recently detached axillary meristems into buds does not occur in many axils of many species.

### 4.7. Summary

The studies above indicate a wide diversity of leaf axil developmental pathways are present in the conifers: (1) bud or meristem initiation commences but is terminated early in the developmental sequence, (2) persisting detached meristems are formed but are abscised by periderm formation, (3) long lived axillary meristems develop, (4) buds may form from dedifferentiated axillary cells with the correct hormonal stimulus or (5) well developed buds may form. Further research is needed in this area, especially for conifer species that are known to be resprouters (Table 1), to determine how they initiate and then maintain buds and/or meristems over time. Many of the 28 resprouting genera listed in Table 1 have had no detailed anatomical investigation of leaf axil structure.

## 5. Conifer Micropropagation

Axillary shoot multiplication or proliferation is generally considered more difficult in conifers than in angiosperm trees (e.g., [90,91,92,93]). As axillary shoot multiplication uses small shoots with nodes and internodes as explants, it is another avenue for investigating axillary meristems/buds in conifer leaf axils. Some conifer species where in vitro axillary bud development has been achieved are as follows: *Araucaria* and *Agathis* [94], *Larix occidentalis* [95], *Podocarpus macrophyllus* [96], *Thuja occidentalis* [88], *Cephalotaxus harringtonia* [97], *Juniperus oxycedrus* [90], *Cupressus sempervirens* and *Chamaecyparis lawsoniana* [98], *Podocarpus henkelii* and *Podocarpus elongatus* [99], *Cedrus atlantica* and *Cedrus libani* [100] and *Metasequoia glyptostroboides* [101]. This is a far from exhaustive listing but focusses on studies where axillary bud development was clearly demonstrated. It includes examples from five of the six conifer families and most of the species above had been recorded as having some type of resprouting ability (Supplementary Table S2).

Several authors have attempted to micropropagate various species of the Araucariaceae using small diameter stem segments, including Burrows et al. [94] (9 *Araucaria* spp., 1 *Agathis* sp.), Sehgal et al. [102] (*Araucaria columnaris*), Iritani et al. [60] (*Araucaria angustifolia*), Gough et al. [103] (*Agathis australis*), Sarmast et al. [104] (*Araucaria heterophylla*) and Niu et al. [105] (*Wollemia nobilis*). These explants usually contained several nodes and internodes and buds developed from the axillary meristems. Whereas most axillary buds are externally visible, these araucarian buds seemingly pushed through the epidermis and outer cortex to emerge [60,62,94,106]. Their progenitor meristems appeared to be endogenous but, as indicated above, were exogenous. Often medium to high concentrations of cytokinins, which can lead to enhanced axillary branching in dicotyledonous tree species, led to the formation of distorted buds [94,104,105]. A single bud developed in each axil. Buds did not develop in the leaf axils of the newly formed buds; thus, high rates of proliferation were not achieved. Vascular connections between the developing bud and the central stele were created by dedifferentiation of cortical parenchyma into xylem and phloem [60,94,106]. Thus, while species of the Araucariaceae approach the angiospermous condition of having long-lived bud-forming potential in all leaf axils, these meristems responded differently in vitro.

## 6. Buds/Meristems in Older Stems

While few studies of conifer leaf axil anatomy have been made, there are even fewer anatomical studies of coppice and epicormic buds and specialised organs such as lignotubers. This is probably a reflection of the relatively few axillary buds that form and the short life span of those that do. In addition, few detailed anatomical studies of epicormic buds are available for either angiosperms or gymnosperms.

Fink [107] investigated the anatomy of epicormic buds of five angiosperm and five gymnosperm species (four from the Pinaceae (*Abies*, *Picea*, *Larix* two species) and one from the Taxaceae (*Taxus*)). Buds of *Taxus baccata* were classified as type 1 (‘high-bud’), those of *Abies alba* as type III (‘deep-bud’) and those of *Picea abies, Larix decidua* and *Larix kaempferi* as type IV (‘shoot-germ’). In type 1 a well-formed bud projects beyond the bark surface, in type III a bud is present but is below the general level of the outer bark and in type IV a preventitious meristem is present within the bark but it has lost its bud-like organisation. In the type IV structure, the tip (leaf primordia and apical meristem) of the bud becomes suberised and is cut off by periderm formation, with the bud base able to maintain its meristematic character for many years. In the 10 species examined by Fink the type IV structure was only found in the conifers.

In most angiosperm trees and shrubs fully formed (apical dome and overlapping leaf primordia) epicormic buds are positioned near the bark surface, with a bud trace that extends through the bark and wood to the pith. As the araucarian axillary meristems in small diameter stems are surrounded by cortical tissues, they are subject to tangential growth stresses as the stems age and increase in diameter. To counteract this, cells in the meristems undergo infrequent anticlinal divisions, thus becoming longer or ‘stretched’ in the tangential plane, but little changed in the radial plane or height. This has been shown for *Araucaria angustifolia* [39] (Figure 33c. 1800 μm), *Araucaria cunninghamii* [108] (Figure 8c. 1000 μm in 3–5 cm diameter) and *Wollemia nobilis* [62] (Figure 2E c. 1300 μm, 4–5 cm diameter) (approximate tangential dimension, diameter of stem where given). Burrows [108] provided indirect evidence that multiple buds might be produced along the length of a stretched meristem. With no vascular trace and no direct indication of their location on the bark surface these meristems can only be located while a remnant of the associated leaf base remains attached to the bark surface. When the meristems are induced to form buds, vascular connections are formed by dedifferentiation of cortical cells [60,62,106]. For *Araucaria angustifolia* Fink [39] stated that these meristems, although long-lived, are eventually abscised by rhytidome formation. For *Araucaria angustifolia* [109] and *Araucaria cunninghamii* [110] high percentages of coppicing were reported from 20 to 26-year-old trees, which indicates a considerable longevity for these meristems.

In *Podocarpus drouynianus* (SW W Australia) and *Podocarpus spinulosus* (coastal NSW and southern Qld) a swelling mass of stem tissue, with associated buds, develops in the axils of the cotyledons soon after germination [111]. The authors note that the buds are more obvious than in the lignotubers of most other Australian species (e.g., eucalypts). In *Podocarpus spinulosus* swellings can also develop in higher leaf axils and on the stems of mature plants. The meristems on stem swellings may develop into roots or shoots. The lignotubers can grow to be more than 400 cm in diameter. Plants of *Podocarpus drouynianus* were subjected to a prescribed burn and then all live shoots were counted three months later [112]. These shoots were then cut off at ground level. All new shoots that appeared were counted, then removed on two further occasions (at 6-week intervals). There was almost no new shoot production after the initial removal of resprouts. Chalwell and Ladd considered that fire had released all the dormant lignotuber buds at the one time and a lack of resprouting after clipping was due to depletion of the bud bank. They noted that fire encouraged lignotuber proliferation and that regenerative tissue on the surface of the lignotuber gradually renewed the bud bank, but presented no anatomical details of how this occurred.

In seedlings of *Sequoia sempervirens* exogenous meristems were present in the axils of the two cotyledons [113,114]. Initially these meristems were poorly differentiated (a single superficial cell layer of meristematic appearance) but by 60 days after germination they had produced leaf primordia and a vascular connection to the stele. The formation of collateral accessory buds then occurred, resulting in clusters of buds. After 4 to 5 years, cortical swelling and further bud proliferation had spread distally to envelop the lowermost nodes produced during the first year of growth. The lignotubers can develop into massive swellings with the surface covered in suppressed buds. Lignotubers can also develop from layered branches, so their initiation was not limited to the cotyledonary axils.

*Ginkgo biloba* produces both basal and aerial lignotubers (‘chichi’). The basal lignotubers developed from superficial meristems in axils of the pair of cotyledons [115]. These meristems, while not obvious in embryos, developed into buds in two-week-old seedlings. After 6 to 12 weeks from germination, a well-developed bud (apical dome with overarching leaf primordia) developed, with one or more lateral accessory buds that became embedded in the periderm. At this stage a well-established vascular connection to the central vascular cylinder had been established. These buds could: (i) develop into clusters of dormant buds within the periderm, (ii) form an aerial shoot, usually after damage to the existing shoot system, or (iii) give rise to an elongated positively geotropic lignotuber with numerous supressed buds on its surface. Both basal and aerial lignotubers develop from preventitious buds, however basal lignotubers develop predictably as a normal part of seedling ontogeny while aerial lignotubers are produced unpredictably on the underside of large lateral branches, usually in response to severe trunk or crown damage. Aerial lignotubers can be up to 2 m in length and can form roots and shoots upon contacting the soil [116].

## 7. Resprouting in *Pinus*

*Pinus* is the largest gymnosperm genus with around 110–120 species. As it is of considerable ecological and economic importance, and also has a wide spectrum of resprouting responses and mechanisms, it is investigated in greater depth below.

It has been proposed that large wildfires have occurred from at least 395 Mya, with charcoal appearing in the fossil record from 420 Mya [15]. The origin of the conifers has been estimated at 350-300 Mya [15], with this group having been used to explore the long interaction between plants and fire [15]. *Pinus* has evolved a suite of fire-dependent functional traits [22] such as serotiny, thick bark, self-pruning of lower branches and resprouting. He et al. [22] considered that fire-adapted traits appeared in the Pinaceae from 126 Ma (thick protective bark), with serotiny dating to 89 Ma. Subsequent research indicated that many early conifers were serotinous in response to intense crown fires from 332 Ma [15].

Various tabular summaries of *Pinus* regeneration strategies have been compiled (each covering 20–113 species), including resprouting (e.g., [117] (Table 12.1), [118] (Table 2), [119] (Table 20.2), [22] (Table S1), [15] (Table S3), [120] (Table S1), [121] (Table 1)). Resprouting was assessed in different ways (e.g., ‘grass stage’, ‘resprouting’, ‘resprouting (young trees)’, ‘recovery from crown scorch’, ‘epicormic’, ‘basal resprouting’) which complicates direct comparisons. The percentage of species with some form of resprouting varied from 40 to 50% when tropical and warm temperate species were assessed (e.g., [118,119]), and 15–25% for studies of 100 plus species (e.g., [22,120]). From the detailed information in the USDA FEIS about 15% of the 39 *Pinus* species of the USA have resprouting capacity. Note that these percentages include seedlings and young trees. Few mature *Pinus* trees can resprout basally after topkill in a fire or after coppicing.

These studies show that a variety of strategies or features can be assessed to determine whether a *Pinus* species is a post-fire resprouter. *Pinus* species range from not being capable of resprouting after fire at any stage of their life cycle to ‘the most fire-resistant pine in the world’ (*Pinus canariensis*) [122]. Supplementary Table S3 indicates conflicting information for several species, e.g., *Pinus clausa*, *Pinus halepensis*, *Pinus heldreichii* and *Pinus occidentalis.* This again shows resprouting capability is not a black and white assessment but depends on fire conditions and plant growth stage, among other factors. Excluding Grivet et al. [123], each study listed 13–24 *Pinus* species with some form of vegetative regeneration. In the present study, 25 species were identified as resprouters. Interestingly all these species are in the subgenus *Pinus* (hard pines), with none in the subgenus *Strobus* (soft pines). It would appear that less than 20% of the 110–120 *Pinus* species have some type of post-fire vegetative regeneration. In many of these species resprouting capability is only present in the juvenile/seedling/sapling stage [68,117].

Layering is quite common in several conifers but is quite rare in *Pinus* [124]. It has been described in *Pinus strobus* [124], *Pinus pumila* [125], *Pinus* x *pseudopumilo* [126] and *Pinus mugo* [127].

As noted above the genus has evolved a range of resprouting strategies and structures, including: (i) grass stage in seedlings, (ii) development of a basal crook, (iii) root collar sprouts and epicormic buds and (iv) protected terminal buds. These are further addressed below.

### 7.1. Grass Stage

The grass stage is probably best investigated in *Pinus merkusii* (SE Asia) and *Pinus palustris* (SE USA) and usually occurs in environments with frequent, low intensity fires. Seedlings with this adaptation have delayed stem elongation for the first five to 10 years after germination [128], with the terminal bud staying at or near ground level [129,130]. During this stage the needles gather as a mantle or dense tuft over the apex [22], thus protecting the shoot apex from the heat of a fire. When “the tufts burn from the needle tips towards the bud, moisture is vaporised which dissipates heat and extinguishes the combustion of the needles” [119] (p. 566). In *Pinus merkusii* seedlings the needles are described as fire-resistant [131], while in *Pinus palustris* the outer layer of needles are destroyed during a fire but the inner part remains intact [132]. During this period of reduced stem elongation a large, deep taproot with substantial carbohydrate stores develops [117,129]. In *Pinus merkusii* a large percentage of the root is cortex and phloem rather than xylem [131]. In *Pinus palustris* when the root collar diameter reaches about 25 mm, the seedlings commence rapid height growth [133]. Eventually this growth elevates the apical meristem above the reach of surface fires. The zone between 0.3 and 1.5 m above ground level is the most critical for bud survival as above 1.5 m the terminal bud can usually survive surface fires [13,134]. The 1.5 m increase in height can occur within 3 years [117]. Having a grass stage does not ensure fire survival as the apical meristem of small seedlings of *Pinus palustris* can be killed by fire. In a post-fire study of 294 seedlings, 50% of plants had died, in 27% the apical meristem survived and in the remaining 23% the apical meristem was killed but the seedlings had resprouted from the root collar [133]. The capacity to resprout from the root collar is an important secondary mechanism for seedling survival but is lost by the sapling stage [135].

The grass stage is relatively uncommon (4 of 35 Mexican pines [118], 8 of 39 tropical taxa [119], 10 of 101 taxa worldwide [22], 13–15 species this study (Supplementary Table S3), chiefly from SE Asia and Central America). It is recorded in four of the six subsections of subgenus *Pinus*. He et al. [22] (p. 755) note the “fire-tolerant grass stage and ability to resprout, are rare and clearly derived among pines” and lineages with these traits were traceable to <40 Ma.

### 7.2. Basal Crook

A basal crook has been recorded in small number of *Pinus* species. It is well developed and extensively described in *Pinus rigida* and *Pinus echinata* and also mentioned as occurring in *Pinus pungens* [136] and *Pinus serotina* [137]. For *Pinus echinata* the basal crook protects dormant basal buds from fire and facilitates resprouting after top kill from fire [138]. In seedlings with a basal crook around 25–75 mm of stem at the base of the seedling (a combination of hypocotyl and epicotyl) forms parallel to and close to ground level before growing upright, thus forming an ‘S’ or a ‘J’ shape [68,139]. Thus, root collar buds in the section of horizontal stem are at ground level and may become covered by soil movement. If so, they would be insulated from the heat of fires [68]. As stem diameter increases the external evidence of the crook disappears [139]. Stone and Stone [68] note that in *Pinus echinata* the crook is almost always present in nursery stock and naturally regenerated seedlings, while Little and Somes [75] note that shade-grown plants have a much higher percentage with poorly developed crooks.

*Pinus taeda* is generally considered a poor resprouter, after both coppicing and fire [140]. When seedlings were cut above the cotyledons 48–72% resprouted but resprouting capacity rapidly decreases with age (at over 8 years of age it was 0%). *Pinus echinata* and *Pinus taeda* hybridise, with the hybrid seedlings having an intermediate development of the basal crook and an intermediate success in resprouting after fire [141,142].

### 7.3. Root Collar Sprouts and Epicormic Buds

Poulos et al. [121] listed fire-adapted traits for about 40 *Pinus* species. They indicated 13 species were basal resprouters and 11 species were epicormic resprouters; this was the only comparative study (see previously) to make this distinction. As mentioned previously *Pinus* seedlings can form buds in the axils of the primary needles just above the cotyledons. The percentage of axils that have buds and those that are blank varies between species and distance up the seedling stem. Of the buds that do form, some elongate soon after initiation while others remain dormant. It is the dormant buds that give rise to root collar sprouts [68]. Early reports often indicated that the root collar sprouts were adventitious but subsequent research showed that their precursors were axillary buds (i.e., preventitious) [68]. In *Pinus roxburghii* seedlings dormant buds are found in the thickened cortex of the stem base [143].

Stone and Stone [144] described the dormant buds of several species (e.g., *Pinus echinata*, *Pinus leiophylla*, *Pinus palustris*, *Pinus rigida*), specifically excluding buds associated with the root collar and the dwarf shoots. These dormant buds were most often found in multinodal pines, where in a single season’s growth there are alternating sections of the stem that have scales that subtended either needle fascicles or scales that were empty or sterile. The uppermost scales of each region with fascicles can bear buds that can develop into normal branches, while others have limited development or remain completely dormant. The dormant buds can become well protected by being concealed within the bark. Following fires that kill or sometimes consume the foliage, the branches of limited growth and the dormant buds can vigorously resprout. Little and Somes [75] made similar observations for *Pinus echinata* and *Pinus rigida*.

### 7.4. Protected Terminal Buds

Regelbrugge and Conard [145] (p. 145) noted that “crown scorch has been implicated as the primary cause of post-fire mortality in conifers, largely due to killing of buds”. Needle kill from scorch does not necessarily equate to crown kill as trees can survive total defoliation [146]. It is possible to have species where 100% crown scorch (foliage killed) is associated with little crown kill (light damage to buds), while there are other species where 100% crown scorch equates to bud and crown kill [147,148]. Bud kill is more important than foliage scorch to tree survival [149] and resistance to heat varies with bud size, with large or shielded buds having greater heat resistance.

While bud size in *Pinus* is important and it is known that certain species have larger buds, there appears to have been little quantification of bud dimensions. In constructing ecological groups for 34 North American *Pinus* species, McCune [150] considered there was insufficient data for several characters of interest, including winter bud size. Tapias et al. [151] investigated terminal bud thickness (width) of seven *Pinus* species in Spain. Average bud width (mm) of the species was 5.0, 5.5, 5.5, 6.5, 8.2, 8.5, 11.5 (mean 7.2, median 6.5). The largest buds were those of *Pinus canariensis*, which was the only species of the seven scored as having resprouting capability. In a study of heat resistance the needles and buds of *Pinus pinea* and *Pinus halepensis* were immersed in hot water for 15 min [152]. The lethal temperature was 10 °C higher for buds than for needles for *Pinus pinea* and 5°C higher for buds than for needles for *Pinus halepensis*. In general, *Pinus halepensis* has a greater fire sensitivity than *Pinus pinea*.

*Pinus* species with large buds and/or shielded by thick (heat resistant) scales and/or long needles include *Pinus elliottii* [153], *Pinus jeffreyi* [118,147,148], *Pinus palustris* [153,154], *Pinus pinaster* [155,156] and *Pinus ponderosa* [147,148,154,157].

## 8. Resprouting after Fire

As noted, this review mainly focuses on resprouting after substantial damage such as coppicing and fire, but also mentions responses to what are usually less intense forms of disturbance such as light pruning, wind damage, insect damage, frost and drought. It also includes some details of other forms of vegetative reproduction, such as layering, which does not directly involve resprouting. All forms of disturbance and subsequent resprouting are of interest in gymnosperms as they indicate the presence of a long-lived bud reserve. However, the disturbance of greatest interest, particularly from an ecological perspective, is fire. Determining if a species can be classified as a post-fire resprouter is not necessarily straightforward as fires can be of different intensities and durations and species can have different responses at different stages of growth (seedling vs. adult) and environmental constraints (e.g., drought). Fire ecologists have limited the definition of a post-fire resprouter to those species that resprout after 100% leaf scorch. Many fires in Northern American pine forests fires produce less than 100% canopy scorch. In addition, many conifers grow in environments where fires are rare and study of post-fire resprouting response is even rarer. Thus, for many species it is difficult to assess if they are post-fire resprouters according to the above definition.

The cycads are a major part of gymnosperm species diversity (c. 350 species). While seedlings can be killed by fire, for most adult plants the columnar stem or caudex is protected by persistent leaf bases [158], with the terminal shoot apical meristem protected by numerous overlapping leaf bases. This is similar to a range of palms, tree ferns and grass trees. These species are classified as apical sprouters, not resprouters per se [36]. Some cycads have subterranean, tuberous fleshy stems [159]. These species can feature root and stem contraction which pulls the shoot apex below ground level, thus protecting it during a fire [159].

In the Ephedraceae many species of *Ephedra* have rhizomes and species such as *Ephedra nevanensis*, *Ephedra torreyana* and *Ephedra viridis* can produce numerous sprouts after top-kill (USDA FEIS).

Many of the species identified in this study and He et al. [15] would probably not be classified as post-fire resprouters. For example, it appears that all 37 species of the Araucariaceae would probably be resprouters after coppicing or severe pruning but few would probably be classified as post-fire resprouters. Possibly the best post-fire resprouter in the family would be *Araucaria araucana*. With extremely thick bark and shedding of lower branches it is well adapted to low intensity fires [160]. Burns [58] noted the continued growth of terminal buds in burnt crowns, as well as epicormic buds on trunks and branches. Root suckers have also been recorded. During the 2019–2020 NSW fire season most *Wollemia nobilis* trees < 8 m tall suffered 100% crown scorch [59]. Although above-ground tissues were killed most plants commenced basal resprouting.

In the Cupressaceae there are resprouting species for which there would appear to be little fire response information (e.g., *Cryptomeria japonica*), species very vulnerable or intolerant of fire even though they form root suckers (e.g., *Athrotaxis cupressoides*, *Diselma archeri*), species able to survive 90–95% but not 100% crown scorch (e.g., *Sequoiadendron giganteum* [157]) and post-fire resprouters (e.g., *Juniperus deppeana*, *Juniperus pinchotii*, *Juniperus oxycedrus, Sequoia sempervirens*, *Taxodium distichum* [161], *Tetraclinis articulata*, *Widdringtonia nodiflora*).

The Pinaceae would appear to have only two post-fire resprouting genera, Pseudotsuga (Pseudotsuga macrocarpa) and Pinus (e.g., *Pinus echinata*, *Pinus elliottii*, *Pinus leiophylla*, *Pinus palustris*, *Pinus pungens*, *Pinus rigida*, *Pinus serotina*, *Pinus canariensis*, *Pinus yunnanensis*).

In the Podocarpaceae *Halocarpus bidwillii* can form basal resprouts after fire but it is not clear if this is after 100% scorch. As in the Cupressaceae there are a couple of genera (e.g., *Microcachrys*, *Pherosphaera*) where clonality (buried trunks, layering, root sprouts) is an important part of population structure, but the plants are highly sensitive to fire. *Podocarpus drouynianus*, *Podocarpus elongatus* and *Podocarpus spinulosus* are accomplished post-fire resprouters.

In the Taxaceae several species of *Taxus* are strong resprouters but not after fire. *Torreya californica* and probably *Torreya taxifolia* resprout after top-kill from fire.

At a world-wide level, the post-fire response after 100% leaf scorch is not known for many conifers. In contrast, detailed information, usually from multiple studies at a range of fire intensities, is given in the USDA FEIS for 94 conifer species for North America. Of these 94 species about 20 could be considered as post-fire resprouters after 100% leaf scorch of adult plants. Post-fire resprouting species include *Juniperus deppeana*, *Juniperus pinchotii*, *Sequoia sempervirens*, *Sequoidendron giganteum*, *Torreya californica*, *Torreya taxifolia*, *Pseudotsuga macrocarpa*, *Taxodium distichum*, *Taxodium mucronatum*. Strong resprouters, but killed by fire, include *Taxus brevifolia* and *Taxus floridana*.

Of the 94 species about 40% are species of *Pinus*. Very few *Pinus* species can survive crown fire as there is little or no basal resprouting in mature trees, while some species can survive after substantial crown scorch [162]. This is substantially different to angiosperms where post-fire resprouters usually resprout from the base, rather than on the stem or branches. Assessing which *Pinus* species can resprout after substantial crown scorch can be quite subjective. It usually occurs in species with thick bark and self-pruning of lower branches (i.e., measures to keep fire out of the canopy). Open crowns, large buds, thick bud scales and tight needle bundles are also associated with resprouting as they can result in a zone where the needles are scorched but the buds are alive. Species where mature plants have a greater degree of tolerance of crown scorch and/or basal resprouting include *Pinus arizonica*, *Pinus echinata*, *Pinus elliottii*, *Pinus jeffreyi*, *Pinus leiophylla*, *Pinus palustris*, *Pinus ponderosa*, *Pinus pungens*, *Pinus resinosa*, *Pinus rigida*, *Pinus serotina* and *Pinus taeda*. In short, compared to angiosperms very few *Pinus* species can resprout after 100% crown scorch and especially after 100% crown consumption, primarily related to poor basal sprouting in mature trees.

Thus, at a world-wide level 11 genera and 24 species (c. 40% *Pinus* species) of conifers have been recorded as resprouting after 100% leaf scorch. The few conifer species, excluding *Pinus* species, that resprout after 100% leaf scorch are mainly from crown-fire ecosystems, although some species (e.g., *Wollemia nobilis*, *Sequoia sempervirens*, *Taxodium distichum*) occur in moist environments. Although there are several resprouting *Pinus* species they require a specific fire intensity to produce leaf scorch (foliage killed by hot gases but not consumed) rather than leaf consumption (foliage is consumed by flaming combustion or is charred). Most of the other resprouting conifer species can resprout after top-kill.

## 9. Conclusions

As indicated in Supplementary Table S1, gymnosperms are widely reported to be poor resprouters, especially when compared to woody angiosperms. Has this review supported the prevailing view? In general, it has, with about 70% of genera not having any species reported to resprout after substantial damage. However, a much wider and more extensive range of genera were found to have resprouting species than would be expected based on the information in Supplementary Table S1.

Resprouting requires buds, protection for those buds and resources to support bud growth. Is the lack of gymnosperm resprouting related to a lack of buds? A wide range of conifers has been reported to have blank or empty leaf axils (no macroscopically visible buds). In the Araucariaceae long-lived detached axillary meristems were consistently present in all the apparently blank leaf axils. In other conifer species, while earlier research indicated the blank axils were devoid of meristems, more recent observations suggest that detached meristems might initially be present in a high percentage of axils but they often have a limited development and life span. Understanding this initial differentiation of axillary meristems and buds in gymnosperms and their subsequent ontogeny will be critical to understanding the resprouting capacity of seedlings, saplings and mature trees.

Condamine et al. [2] considered that increased extinction of gymnosperms was related to increased competition from angiosperms. This has been related to a wide diversity of morphological, anatomical, physiological and genetical factors. Perhaps the greater abundance and longevity of angiosperm axillary buds and hence greater resprouting capacity is another part of their competitive advantage.

## Figures and Tables

**Table 1:** Gymnosperm families and genera with at least one ‘strongly’ resprouting species, based on information in Supplementary Table S2. The number in brackets after the family name indicates the number of genera in that family.

Family	Genera
non-conifers	
Cycadaceae (1)	
Zamiaceae (9)	*Encephalartos*
Ginkgoaceae (1)	*Ginkgo*
Ephedraceae (1)	*Ephedra*
Gnetaceae (1)	*Gnetum*
Welwitschiaceae (1)	-
conifers	
Araucariaceae (3)	*Agathis*, *Araucaria*, *Wollemia*
Cupressaceae (30)	*Actinostrobus*, *Cryptomeria*, *Cunninghamia*, *Cupressus*, *Juniperus*, *Sequoia*, *Sequoiadendron*, *Taxodium*, *Tetraclinis*, *Widdringtonia*
Pinaceae (11)	*Keteleeria*, *Pinus*, *Pseudotsuga*
Podocarpaceae (19)	*Halocarpus*, *Manoao*, *Phyllocladus*, *Podocarpus*
Sciadopityaceae (1)	-
Taxaceae (6)	*Amentotaxus*, *Cephalotaxus*, *Taxus*, *Torreya*

## Data Availability

Data is contained within the article and the supplementary file.

## Supplementary Materials

The following are available online at https://www.mdpi.com/article/10.3390/plants10122551/s1, Supplementary Table S1. Various quotations regarding the resprouting capacity of gymnosperms, arranged in chronological order. Supplementary Table S2. Vegetative reproduction in gymnosperms. Supplementary Table S3. *Pinus* species that have been reported to be capable of vegetative reproduction.
