# Supplementary material for: Gymnosperm Resprouting—A Review
Source: Plants (Basel). 2021 Nov 23;10(12):2551. doi: 10.3390/plants10122551 (PMC8705048; doi:10.3390/plants10122551)
Supplement: Supplementary file 1 [file plants-10-02551-s001.zip › Supplementary plants-1387177/Table S1 quotes.pdf]

Supplementary table S1: Various quotations regarding the resprouting capacity of gymnosperms, arranged in chronological order.

References	Quotations regarding resprouting
Lust and Mohammady (1973 p. 12)	"Little is known about the sprouting capacity of conifers. In most European handbooks it is even stated that conifers do not possess the ability to sprout. Several coniferous species, however, are known to sprout …"
Evans (1992 p. 18)	"Most broadleaved species will coppice, most conifers will not, with the main exceptions of …"
Bellingham et al. (1994 p. 754)	"… conifers generally sprout less frequently than angiosperms …"
Loehle (2000 p. 18)	"Many species, particularly most conifers, do not sprout or sprout poorly, …"
Bond and Midgley (2001 p. 45)	"Most conifers do not sprout although there are exceptions in several unrelated lineages, including species of *Pinus* in the north and *Podocarpus* in the southern hemisphere."
Del Tredici (2001 p. 121)	"Sprouting is a universal attribute of temperate angiosperm trees through the sapling stage of development but is much less common among gymnosperms."
Lanner (2002 p. 657)	"Epicormic branches are virtually ubiquitous in broadleaved trees, and have been considered relatively uncommon in conifers."
Meier et al. (2012 p. 575)	"Conifers, which in general produce fewer epicormic sprouts on the bole …"
Clarke et al. (2013 p. 24)	"By contrast with angiosperms, epicormic resprouting in gymnosperms is limited to a few species (e.g. *Pinus canariensis*), …"
Wendling et al. (2014 p. 479)	"Many tree species, conifers in particular, produce few or no coppice shoots upon felling …"
Zeppel et al. (2015 p. 584)	"There are fewer gymnosperms that resprout, although some species within *Pinus*, *Juniperus* and *Abies* do."
Buckley and Mills (2015 p. 77-78)	"Most woody broadleaves coppice freely, at least when young." "Conifers lack basal buds and may sprout only weakly if damaged, with some exceptions …"
McDowell and Allen (2015 p.670)	"Coniferous trees also commonly lack the ability to epicormically resprout after mortality of their aboveground tissues relative to many angiosperm trees and shrubs."
Pausas and Keeley (2017 p. 1010)	"Conifers are distinct in that resprouting is relatively rare compared with woody angiosperms. A few species do resprout …; however, they generally do not resprout epicormically after an intense fire, with a few exceptions."
Keeley et al. (2019 p. 775)	"The vast majority of gymnosperm species lack the capacity to regenerate by resprouting from burned stems (with some notable exceptions)."

Bellingham PJ, Tanner EVJ, Healey JR. 1994. Sprouting of trees in Jamaican montane forests, after a hurricane. *Journal of Ecology,* **82**: 747-758.

Bond WJ, Midgley JJ. 2001. Ecology of sprouting in woody plants: the persistence niche. *Trends in Ecology and Evolution,* **16**: 45-51.

Buckley P, Mills J. 2015. Coppice silviculture: from the Mesolithic to the 21st Century. In: Kirby KJ, Watkins C, eds. *Europe's changing woods and forests. From wildwood to managed landscapes.* Wallingford: CABI.

Clarke PJ, Lawes MJ, Midgley JJ, Lamont BB, Ojeda F, Burrows GE, Enright NJ, Knox KJE. 2013. Resprouting as a key functional trait: how buds, protection and resources drive persistence after fire. *New Phytologist,* **197**: 19-35.

Del Tredici P. 2001. Sprouting in temperate trees: A morphological and ecological review. *Botanical Review,* **67**: 121-140.

Evans J. 1992. Coppice forestry - an overview. In: Buckley GP, ed. *Ecology and management of coppice woodlands*. London: Chapman and Hall.

Keeley JE, van Mantgem P, Falk DA. 2019. Fire, climate and changing forests. *Nature Plants,* **5**: 774-775.

Lanner RM. 2002. Why do trees live so long? *Ageing Research Reviews,* **1**: 653-671.

Loehle C. 2000. Strategy space and the disturbance spectrum: A life-history model for tree species coexistence. *American Naturalist,* **156**: 14-33.

Lust N, Mohammady M. 1973. Regeneration of coppice. *Silva Gandavensis,* **39**: 1-28.

McDowell NG, Allen CD. 2015. Darcy's law predicts widespread forest mortality under climate warming. *Nature Climate Change,* **5**: 669-672.

Meier AR, Saunders MR, Michler CH. 2012. Epicormic buds in trees: a review of bud establishment, development and dormancy release. *Tree Physiology,* **32**: 565-584.

Pausas JG, Keeley JE. 2017. Epicormic resprouting in fire-prone ecosystems. *Trends in Plant Science,* **22**: 1008-1015.

Wendling I, Trueman SJ, Xavier A. 2014. Maturation and related aspects in clonal forestry-part II: reinvigoration, rejuvenation and juvenility maintenance. *New Forests,* **45**: 473-486.

Zeppel MJB, Harrison SP, Adams HD, Kelley DI, Li G, Tissue DT, Dawson TE, Fensham R, Medlyn BE, Palmer A, West AG, McDowell NG. 2015. Drought and resprouting plants. *New Phytologist,* **206**: 583-589.
