# Supplementary material for: Gymnosperm Resprouting—A Review
Source: Plants (Basel). 2021 Nov 23;10(12):2551. doi: 10.3390/plants10122551 (PMC8705048; doi:10.3390/plants10122551)
Supplement: Supplementary file 1 [file plants-10-02551-s001.zip › Supplementary plants-1387177/Table S2 gymnosperms.pdf]

Supplementary table S2. Vegetative reproduction in gymnosperms. The table concentrates on resprouting after substantial disturbance but also includes some information on epicormic shoot formation as a normal part of crown maintenance and layering. Species numbers per genus from The Plant List 1.1 (www.theplantlist.org). In larger genera where several species are listed below it is probable that other species in these genera would be capable of vegetative reproduction. In the references 'USDA FEIS' stands for the United States Department of Agriculture Fire Effects Information System (https://www.feis-crs.org/feis/) which was mainly accessed during January-February 2017.

Family/Genus	Comments	Reference
Subclass Cycadidae		
**Cycadaceae**		
Cycas (92)	Adventitious buds/branches common in many *Cycas* species, especially after damage	Stevenson (2020)
	C. micronesica, C. seemannii: snapped trees after cyclone damage – adventitious shoots formed on stump, detached apical part of stem formed adventitious roots	Keppel (2001), Marler and Lawrence (2013)
**Zamiaceae**		
9 genera (216)	Many genera form adventitious buds after decapitation or other wounding	Stevenson (2020)
	Encephalartos species: basal suckering is common, while production of aboveground adventitious branches as plant matures or after severe damage is not as frequent	Cousins and Witkowski (2017)
Subclass Ginkgoidae		
**Ginkgoaceae**		
Ginkgo (1)	*G. biloba*: clonal regeneration by basal chichi (lignotuber or burl) and aerial chichi	Del Tredici (1992, 1997)
Subclass Gnetidae		
**Ephedraceae**		
Ephedra (70)	*E. nevadensis, E. torreyana, E. viridis*: resprouting from roots, woody crown and stump tissues; for *E. torreyana* hundreds of stump sprouts per shrub can develop after fire	Parmenter (2008), USDA FEIS
	Numerous species possess rhizomes	McLean (1950)

	E. nevadensis layering and underground stems	Land (1913)
**Gnetaceae**		
Gnetum (41)	*G. africanum* and *G. buchholzianum*: lianas that can regenerate from buds on underground tubers and from root suckers	Shiembo (1999), Ali et al. (2011), Caspa et al. (2014), Biye et al. (2017)
	G. gnemon can reiterate trunk axes from reserve axillary buds	Tomlinson (2001)
**Welwitschiaceae**		
Welwitschia (1)	*W. mirabilis*: no resprouting but the two leaves can regenerate from a basal meristem	Brinckmann and von Willert (1987)
Subclass Pinidae		
**Araucariaceae** (38)		
Agathis (18)	*A. australis* (NZ) coppicing	Ecroyd (1982), Perry et al. (2014), Teixeira et al. (2020)
	A. borneensis (Malaysia) saplings will coppice	Thomson (2006)
	A. dammara (SE Asia) coppice shoots	Momose (1978)
	A. robusta (Aust.) coppicing and root buds	Haley (1957), Nikles (1961)
Araucaria (19)	Reiteration is widespread in the genus	Veillon (1978)
	A. angustifolia (S. Amer.) coppice from cut stumps	Wendling et al. (2009), Alabarce and Dillenburg (2012), Wendling and Brondani (2015)
	A. araucana (S. Amer.) stump sprouts and root suckers; epicormic shoots	Veblen (1982), Burns (1993), Farjon (2010)
	A. cunninghamii (Aust.) coppice from cut stumps; root buds on damaged roots	Nikles (1961), Burrows (1990a, 1990b), House et al. (1998)
	A. heterophylla (Norfolk Is.) seedlings and young trees resprouted after decapitation	Marin (1961), Schubert and Zambrana (1982)
	A. hunsteinii (Papua New Guinea) 3–5-year-old saplings resprouted after decapitation	Ahmad (1982)
Wollemia (1)	*W. nobilis* (Aust.) coppice and epicormic shoots; aerial resprouts after complete	Hill (1997), Burrows et al. (2003), Zimmer

	defoliation (after fire)	et al. (2015), Prior and Bowman (2020)
**Cupressaceae** (162)		
Actinostrobus (3)	*A. acuminatus* (W. Aust.) epicormic buds on ribbon-like underground stems	Bowman and Harris (1995)
Athrotaxis (3)	*A. cupressoides* (Tas.) fire sensitive but vegetative regeneration from root suckers	Cullen and Kirkpatrick (1988), Worth et al. (2016)
	A. selaginoides (Tas.) rooted shoots from fallen saplings and root suckers	Read and Hill (1988)
Austrocedrus (1)	*A. chilensis* (S. Amer.) no sprouting from stumps or roots but can branch layer; relies exclusively on seed production	Aparicio et al. (2009) Landesmann et al. (2015)
Callitris (15)	All species (mainly Aust.) fire sensitive; larger trees of some species can epicormically resprout after surface fire	Bowman and Harris (1995), Luly (2001), Prior et al. (2007), Haverkamp et al. (2015)
	C. intratropica and *C. glaucophylla* can weakly resprout after canopy fire	Prior and Bowman (2020)
Calocedrus (4)	*C. decurrens* (N. Amer.) no records of coppice or epicormic resprouting.	USDA FEIS
Chamaecyparis (5)	Coppicing or epicormic resprouting not recorded. Branch layering in *C. pisifera* (Jap.) and various N. Amer. species. Seedling stems of *C. pisifera* can resprout after browsing	Cooper (1911), Yamamoto et al. (1994), Yamamoto (1998), Hayakawa et al. (2004), USDA FEIS
Cryptomeria (1)	*C. japonica* (Japan) extensive layering especially at higher altitudes	Taira et al. (1997), Hirayama and Sakimoto (2003), Doğan et al. (2017)
	Coppices well and produces root suckers	Savill (2015)
Cunninghamia (2)	*C. lanceolata* (e Asia) prolific stump resprouter	Li and Ritchie (1999), Li et al. (2010), Cheng et al. (2011)
Cupressus (19)	Generally obligate seeders with no resprouting after fire	Zedler (1995), Ne'eman et al. (1999), de Gouvenain and Ansary (2006), USDA FEIS
	C. sempervirens (Mediterranean) resprouts from cut stumps	Couppis (1954)
Diselma (1)	*D. archeri* (Tasmania) fire sensitive, can root sucker and form clonal patches	Kirkpatrick et al. (2010), Worth et al. (2017)
Fitzroya (1)	*F. cupressoides* (S. Amer.) root suckering, layering	Veblen and Ashton (1982), Parker and Donoso (1993), Silla et al. (2002)
Fokienia (1)	SE Asia, unknown	
Glyptostrobus (1)	*G. pensilis* (China) vegetative reproduction recorded, but no details provided	Tang et al. (2019)
Juniperus (75)	Most North American species are non-sprouting. Sprouting species, e.g., *J. pinchotii* and *J. deppeana* (N. Amer.) are strong resprouters after coppicing and	Phillips (1911), Smith et al. (1975), Wood et al. (2012), Rodríguez-Trejo et al. (2019),

	fire; *J. deppeana* can resprout from root-crown, lignotuber, epicormic buds	USDA FEIS
	J. oxycedrus (Mediterranean) lignotuberous resprouter	López-Soria and Castell (1992), Paula et al. (2016)
	J. foetidissima (Mediterranean) coppice shoots	Sagheb-Talebi et al. (2001)
Libocedrus (5)	*L. bidwillii* (NZ) considered non-sprouting but epicormic shoots and layering recorded	Clayton-Greene (1977), Haase (1990), Martin and Ogden (2006)
Metasequoia (2)	*M. glyptostroboides* (China) epicormic shoots in crown	Jagels and Day (2004)
Microbiota (1)	*M. decussata* (Russia) layering	Artyukova et al. (2009), Farjon (2010)
Neocallitropsis (1)	*N. pancheri* (New Caledonia) fire sensitive, resprouting not recorded	McCoy et al. (1999)
Papuacedrus (1)	*P. papuana* (SE Asia) unknown	
Pilgerodendron (1)	*P. uviferum* (S. Amer.) trees killed by fire; layering and root suckering reported	Allnutt et al. (2003), Soto et al. (2007), Bannister et al. (2012)
Platycladus (1)	*P. orientalis* (E. Asia) unknown	
Sequoia (1)	*S. sempervirens* (USA) lignotubers and burls producing coppice and epicormic shoots, layering, root suckers	Lamb (1899), Del Tredici (1998, 1999), Douhovnikoff et al. (2004), O'Hara et al. (2017)
Sequoiadendron (1)	*S. giganteum* (USA) coppicing of stumps of younger trees; epicormic shoots	Piirto et al. (1986), Weatherspoon (1990), Ahuja (2009), USDA FEIS
Taiwania (1)	*T. cryptomerioides* (E. Asia) weak basal sprouting ability	He et al. (2015)
Taxodium (2)	*T. distichum* (USA) prolific stump resprouting	Conner et al. (1986), Randall et al. (2005)
Tetraclinis (1)	*T. articulata* (Mediterranean) lignotubers and regeneration from coppice	Rourke (1991), Farjon (2010), Paula et al. (2016)
Thuja (5)	Generally fire sensitive, not resprouters, epicormic shoots in the canopy; layering	Potzger (1937), Edelstein and Ford (2003), USDA FEIS
	T. sutchuenensis (China) strong resprouting after disturbance	Tang et al. (2015)
Thujopsis (1)	*T. dolabrata* (Japan) layering recorded	Farjon (2010), Hitsuma et al. (2015)
Widdringtonia (4)	*W. nodiflora* (southern Africa) vigorous basal resprouting after fire, *W. wallichii* and *W. schwarzii* (southern Africa) do not resprout	Keeley et al. (1999), Rundel (2019)
Xanthocyparis (2)	*X. nootkatensis* (N. Amer.) layers readily	Ritland et al. (2001), Krapek and Buma (2018), USDA FEIS

**Pinaceae** (247)		
Abies (48)	*A. concolor* and *A. religiosa* (N. Amer.) epicormic shoots after crown scorch	Hanson and North (2006), Temiño-Villota et al. (2016)
	A. balsamea, *A. lasiocarpa* and *A. fraseri* (N. Amer.) layering	Cooper (1911), Bannan (1942), Sirois (1997), Lanner (2002), Holtmeier and Broll (2017), USDA FEIS
Cathaya (1)	*C. argyrophylla* (China) resprouting not noted	He et al. (2012)
Cedrus (3)	Mediterranean, not post-fire resprouters, *C. libani* produces epicormics after severe defoliation by moths	Carus and Avci (2005), Pausas et al. (2008)
Keteleeria (3)	(e Asia) all species can sprout after coppicing	Frankis (1988), Farjon (1989, 2010)
Larix (11)	Mostly no resprouting but *L. occidentalis* (N. Amer.) epicormic shoots after pruning and as a part of normal crown maintenance; *L. laricina* (N. Amer.) layering	Bannan (1942), Lanner (1995), O'Hara and Valappil (2000), Waring and O'Hara (2005), USDA FEIS
	L. gmelinii (Siberia and NE Asia) layering	Kruse et al. (2020)
Nothotsuga (1)	*N. longibracteata* (China) resprouting not noted	He et al. (2012)
Picea (36)	*P. sitchensis* (N. Amer.) produces epicormics after pruning, thinning and wind damage; layering and root sprouts – e.g., *P. engelmannii*, *P. mariana, P. rubens, P. glauca* (N. Amer.)	Cooper (1931), Potzger (1937), Stanek (1968), Del Tredici (2001), Lanner (2002), Deal et al. (2003), Quine (2004), USDA FEIS
Pinus (130)	Of 101 species 16 were noted to have some resprouting capacity	He et al. (2012), see also Supp. Table 2
	Basal sprouts from at least seedlings/young trees: e.g., *P. echinata, P. leiophylla, P. oocarpa, P. rigida, P. serotina*	Stone and Stone (1954), Venator (1977), Lilly et al. (2012), Bradley et al. (2016), USDA FEIS
	Epicormic sprouts after crown scorch: e.g., *P. canariensis*	Climent et al. (2004), Fernandes et al. (2008)
	P. mugo (European) and *P. pumila* (N Asia): layering	Jeník (1994)
	P. yunnanensis (China) has tree and shrub forms. After crown fire the shrub forms basal resprouts	Pausas et al. (2021)
Pseudolarix (1)	*P. amabilis* (China) resprouting not noted	He et al. (2012)
Pseudotsuga (4)	*P. macrocarpa* (N. Amer.) coppice and epicormic shoots after injury/fire	USDA FEIS
	P. menziesii (N. Amer.) epicormic shoots for crown maintenance, no basal or	Bryan and Lanner (1981), Ishii & Ford

	epicormic ability after fire	(2001), Lazzeri-Aerts & Russell (2014)
	Root suckers	Lamb (1899), Potzger (1937)
Tsuga (9)	Four N. Amer. spp. very fire sensitive, no record of coppicing or epicormic shoots; layering relatively common; *T. heterophylla* (N. Amer.) crown maintenance epicormics; *T. canadensis* (N. Amer.) no resprouting after windthrow	Cooper (1931), Peterson and Pickett (1991), Lanner (2002), Del Tredici and Orwig (2017), USDA FEIS
**Podocarpaceae** (191)		
Acmopyle (2)	New Caledonia and Fiji, resprouting not recorded	
Afrocarpus (5)	Africa, resprouting not recorded	
Dacrycarpus (9)	*D. imbricatus* (SE Asia) some basal resprouting from fully grown trees	de Laubenfels (1988)
Dacrydium (21)	*D. cupressinum* (NZ) natural vegetative reproduction not recorded	Franklin (1968), Norton et al. (1988)
	D. xanthandrum (se Asia) root suckers	Wong (1994)
Falcatifolium (6)	*F. taxoides* (N. Caledonia) occasional epicormic shoots	Mill and Thomas (1999)
Halocarpus (3)	*Halocarpus* species (NZ) are fire sensitive, generally not resprouting although *H. bidwillii* basal resprouting after fire; *H. bidwillii* and *H. biformis* vegetative spread through layering	Wardle (1963), Burrows (1994), Coomes and Bellingham (2011), Perry et al. (2014), McGlone et al. (2017)
Lagarostrobos (1)	*L. franklinii* (Tas.) very fire sensitive, epicormic resprouts from fallen trees, layering and suckers	Gibson and Brown (1991), Shapcott (1991), Molloy (1995)
Lepidothamnus (3)	2 spp. NZ, 1 sp. S. Amer. *L. fonkii* (S. Amer.) very dense spreading mats, *L. intermedius* (NZ) vegetative spread	Gardner and Lara (2003), Coomes and Bellingham (2011)
Manoao (1)	*M. colensoi* (NZ) prolific sucker shoots from horizontal underground stems	Molloy (1995)
Microcachrys (1)	*M. tetragona* (Tas.) fire intolerant, resprouts from buried trunks, appears to form ‘clonal swarms’, layering	Kirkpatrick (1984), Booth (2013)
Nageia (6)	*N. nagi* (Asia) some sprouting after felling; ineffective resprouting after typhoon damage	Wu et al. (2008), Coomes and Bellingham (2011)
Parasitaxus (1)	*P. ustus* (New Cal.) parasitic on *Falcatifolium taxoides* – resprouting not recorded	
Pherosphaera (2)	*P. hookeriana* (Tas.) thought to reproduce predominately asexually by root suckering but clonality plays a small role in population level regeneration	Worth et al. (2018, 2021)

Phyllocladus (4)	"whole genus appears to have the property of coppicing vigorously from epicormic shoots"	Page (1990)
	P. alpinus (NZ) spreads vegetatively from underground stems, layering, sprouting after uprooting, epicormic shoots, root sprouts	Wardle (1963), Ogden et al. (1991), Burrows (1994), Molloy (1995)
	P. toatoa (NZ) coppice and epicormic shoots	Molloy (1996)
Podocarpus (*c*. 110)	*P. spinulosus* (E. Aust) woody clumps from lignotuber/stem burl, layering	Lacey and Johnston (1990), Farjon (2010), Ladd and Enright (2011)
	P. drouynianus (W. Aust.) strong resprouter from lignotuber	Chalwell and Ladd (2005), Ladd and Enright (2011)
	P. macrophyllus (Asia) used for hedges and topiary	Tomlinson and Huggett (2011)
	P. nivalis (NZ) – layering	Wardle (1963)
	P. lawrencei (E. Aust.) – root suckers, epicormic shoots	Tolsma et al. (2004), Kirkpatrick et al. (2010)
	P. elongatus (S. Afr.) epicormic buds sprout after total leaf scorch from fire, root sprouts	Midgley et al. (1995)
	P. urbanii (Jamaica) – sprouted after hurricane damage	Bellingham et al. (1994)
Prumnopitys (9)	E. & W. Pacific – vegetative reproduction not recorded	Perry et al. (2014), Teixeira et al. (2020)
Retrophyllum (5)	Papuasia and S. Amer. Fire sensitive, vegetative reproduction not recorded	McCoy et al. (1999)
Saxegothaea (1)	*S. conspicua* (S. Amer.) thin-barked and easily killed by fire; basal and epicormic sprouting	Veblen et al. (1980), Lusk (1996), Veblen et al. (2005)
Sundacarpus (1)	*S. amarus* (Aust., SE Asia) resprouting not recorded	
**Sciadopityaceae** (1)		
Sciadopitys (1)	*S. verticillata* (Japan) layering	Worth et al. (2014)
**Taxaceae** (31)		
Amentotaxus (6)	*A. formosana* (Taiwan) sprouts from trunk base	Lin et al. (2007)
Austrotaxus (1)	*A. spicata* (New Cal.) resprouting not recorded	
Cephalotaxus (8)	*C. harringtonii* (Japan) resprouts from base or roots, resprouts after frost damage	Nowakowska and Baran (2007), Farjon (2010)
Pseudotaxus (1)	*P. chienii* (China) resprouting not recorded	

Taxus (9)	*T. baccata* (Africa/Europe/Asia) strong epicormic and coppice resprouting	Thomas and Polwart (2003)
	T. brevifolia (N. Amer.) basal sprouts from stumps and layering, epicormic shoots	Busing et al. (1995), Minore and Weatherly (1996), USDA FEIS
	T. canadensis (N. Amer.) layering	Allison (1991), Windels and Flaspohler (2011), USDA FEIS
	T. wallichiana (Asia) basal sprouting from stumps and root collars, epicormic sprouting also recorded	Farjon (2010), Zhang et al. (2018)
	T. floridana (Florida, USA) layering, root and stem sprouts	USDA FEIS
Torreya (6)	*T. californica* (USA) sprouts vigorously from cut stumps, root suckers	Potzger (1937), Burke (1975), USDA FEIS
	T. taxifolia (USA) root suckers, basal and epicormic shoots	Schwartz et al. (2000)

References

Ahmad DBH. 1982. Vegetative propagation of *Araucaria hunsteinii* by cuttings. *Malaysian Forester,* **45**: 81-83.

Ahuja MR. 2009. Genetic constitution and diversity in four narrow endemic redwoods from the family Cupressaceae. *Euphytica,* **165**: 5-19.

Alabarce FS, Dillenburg LR. 2012. Resprouting ability and intensity after damage in seedlings of the large-seeded species *Araucaria angustifolia*. *Brazilian Journal of Plant Physiology,* **24**: 131-140.

Ali F, Assanta MA, Robert C. 2011. *Gnetum africanum*: A wild food plant from the African forest with many nutritional and medicinal properties. *Journal of Medicinal Food,* **14**: 1289-1297.

Allison TD. 1991. Variation in sex expression in Canada yew (*Taxus canadensis*). *American Journal of Botany,* **78**: 569-578.

Allnutt TR, Newton AC, Premoli A, Lara A. 2003. Genetic variation in the threatened South American conifer *Pilgerodendron uviferum* (Cupressaceae), detected using RAPD markers. *Biological Conservation,* **114**: 245-253.

Aparicio A, Pastorino M, Martinez-Meier A, Gallo L. 2009. Vegetative propagation of patagonian cypress, a vulnerable species from the subantarctic forest of South America. *Bosque,* **30**: 18-26.

Artyukova EV, Kozyrenko MM, Gorovoy PG, Zhuravlev YN. 2009. Plastid DNA variation in highly fragmented populations of *Microbiota decussata* Kom. (Cupressaceae), an endemic to Sikhote Alin Mountains. *Genetica,* **137**: 201-212.

Bannan MW. 1942. Notes on the origin of adventitious roots in the native Ontario conifers. *American Journal of Botany,* **29**: 593-598.

Bannister JR, Donoso PJ, Bauhus J. 2012. Persistence of the slow growing conifer *Pilgerodendron uviferum* in old-growth and fire-disturbed southern bog forests. *Ecosystems,* **15**: 1158-1172.

Bellingham PJ, Tanner EVJ, Healey JR. 1994. Sprouting of trees in Jamaican montane forests, after a hurricane. *Journal of Ecology,* **82**: 747-758.

Biye EH, Balkwill K, Cron GV. 2017. Taste versus shelf life: Intended use should guide selection of indigenous strains of *Gnetum* L. (Gnetaceae) for domestication in Africa. *South African Journal of Botany,* **113**: 170-181.

Booth C. 2013. Enduring Australians millenarians and centenarians. *Wildlife Australia,* **50(3)**: 4-9.

Bowman DMJS, Harris S. 1995. Conifers of Australia's dry forests and open woodlands. In: Enright NJ, Hill RS, eds. *Ecology of the southern conifers.* Melbourne: Melbourne University Press.

Bradley JC, Will RE, Stewart JF, Nelson CD, Guldin JM. 2016. Post-fire resprouting of

shortleaf pine is facilitated by a morphological trait but fire eliminates shortleaf x loblolly pine hybrid seedlings. *Forest Ecology and Management,* **379**: 146-152.

Brinckmann E, von Willert DJ. 1987. Injury and recovery of *Welwitschia mirabilis*. *Dinteria,* **19**: 69-76.

Bryan JA, Lanner RM. 1981. Epicormic branching in Rocky Mountain Douglas-fir. *Canadian Journal of Forest Research,* **11**: 190-199.

Burke JG. 1975. Human use of the California nutmeg tree, *Torreya californica*, and other members of the genus. *Economic Botany,* **29**: 127-139.

Burns BR. 1993. Fire-induced dynamics of *Araucaria araucana - Nothofagus antarctica* forest in the southern Andes. *Journal of Biogeography,* **20**: 669-685.

Burrows C. 1994. Do New Zealand forest trees regenerate from sprouts? *Canterbury Botanical Society Journal,* **28**: 63-68.

Burrows GE. 1990a. The role of axillary meristems in coppice and epicormic bud initiation in *Araucaria cunninghamii*. *Botanical Gazette,* **151**: 293-301.

Burrows GE. 1990b. Anatomical aspects of root bud development in hoop pine (*Araucaria cunninghamii*). *Australian Journal of Botany,* **38**: 73-78.

Burrows GE, Offord CA, Meagher PF, Ashton K. 2003. Axillary meristems and the development of epicormic buds in Wollemi pine (*Wollemia nobilis*). *Annals of Botany,* **92**: 835-844.

Busing RT, Halpern CB, Spies TA. 1995. Ecology of Pacific yew (*Taxus brevifolia*) in Western Oregon and Washington. *Conservation Biology,* **9**: 1199-1207.

Carus S, Avci M. 2005. Growth loss of Lebanon cedar (*Cedrus libani*) stands as related to periodic outbreaks of the cedar shoot moth (*Dichelia cedricola*). *Phytoparasitica,* **33**: 33-48.

Caspa RG, Tchouamo IR, Mweru JM, Amang JM. 2014. The ecological status and uses of *Ricinodendron heudelotii* (Baill.) Pierre and *Gnetum* species around the Lobeke National Park in Cameroon. *Agriculture, Forestry and Fisheries,* **3**: 469-480.

Chalwell STS, Ladd PG. 2005. Stem demography and post fire recruitment of *Podocarpus drouynianus*: a resprouting non-serotinous conifer. *Botanical Journal of the Linnean Society,* **149**: 433-449.

Cheng X, Kiyoshi U, Tsuyoshi H, Shao P. 2011. Height growth, diameter-height relationships and branching architecture of *Pinus massoniana* and *Cunninghamia lanceolata* in early regenerartion stages in Anhui Provine, eastern China: effects of light intensity and regeneration mode. *Forestry Studies in China,* **13**: 1-12.

Clayton-Greene KA. 1977. Structure and origin of *Libocedrus bidwillii* stands in the Waikato District, New Zealand. *New Zealand Journal of Botany,* **15**: 19-28.

Climent J, Tapias R, Pardos JA, Gil L. 2004. Fire adaptations in the Canary Islands pine (*Pinus canariensis*). *Plant Ecology,* **171**: 185-196.

Conner WH, Toliver JR, Sklar FH. 1986. Natural regeneration of baldcypress (*Taxodium distichum* (L.) Rich.) in a Louisiana swamp. *Forest Ecology and Management,* **14**: 305-317.

Coomes DA, Bellingham PJ. 2011. Temperate and tropical podocarps: how ecologically alike are they? In: Turner BL, Cernusak LA, eds. *Ecology of the Podocarpaceae in tropical forests.* Washington DC: Smithsonian Institution Scholarly Press.

Cooper WS. 1911. Reproduction by layering among conifers. *Botanical Gazette,* **52**: 369-379.

Cooper WS. 1931. The layering habit in Sitka spruce and the two western hemlocks. *Botanical Gazette,* **91**: 441-451.

Couppis TA. 1954. Some notes on the Mediterranean cypress, *Cupressus sempervirens*, found in Cypress. *Empire Forestry Review,* **33**: 71-73.

Cousins SR, Witkowski ETF. 2017. African cycad ecology, ethnobotany and conservation: a synthesis. *Botanical Review,* **83**: 152-194.

Cullen PJ, Kirkpatrick JB. 1988. The ecology of *Athrotaxis* D. Don (Taxodiaceae). I. Stand structure and regeneration of *A. cupressoides*. *Australian Journal of Botany,* **36**: 547-560.

De Gouvenain RC, Ansary AM. 2006. Association between fire return interval and population dynamics in four California populations of Tecate cypress (*Cupressus forbesii*). *Southwestern Naturalist,* **51**: 447-454.

de Laubenfels DJ. 1988. Coniferales. *Flora Malesiana*. Dordrecht: Kluwer Academic.

Deal RL, Barbour RJ, McClellan MH, Parry DL. 2003. Development of epicormic sprouts in Sitka spruce following thinning and pruning in south-east Alaska. *Forestry,* **76**: 401-412.

Del Tredici P. 1992. Natural regeneration of *Ginkgo biloba* from downward growing cotyledonary buds (basal chichi). *American Journal of Botany,* **79**: 522-530.

Del Tredici P. 1997. Lignotuber development in *Ginkgo biloba*. In: Hori T, Ridge RW, Tulecke W, Del Tredici P, Trémouillaux-Guiller J, Tobe H, eds. *Ginkgo biloba - a global treasure.* Tokyo: Springer-Verlag.

Del Tredici P. 1998. Lignotubers in *Sequoia sempervirens*: development and ecological significance. *Madroño,* **45**: 255-260.

Del Tredici P. 1999. Redwood burls: immortality underground. *Arnoldia,* **59**: 14-22.

Del Tredici P. 2001. Sprouting in temperate trees: A morphological and ecological review. *Botanical Review,* **67**: 121-140.

Del Tredici P, Orwig DA. 2017. Layering and rejuvenation in *Tsuga canadensis* (Pinaceae) on Wachusett Mountain, Massachusetts. *Rhodora,* **119**: 16-32.

Doğan G, Tadama T, Kohama H, Matsumoto A, Moriguchi Y. 2017. Evidence of clonal

propagation in *Cryptomeria japonica* D. Don distributed on Pacific Ocean side in Japan. *Silvae Genetica,* **66**: 43-46.

Douhovnikoff V, Cheng AM, Dodd RS. 2004. Incidence, size and spatial structure of clones in second-growth stands of coast redwood, *Sequoia sempervirens* (Cupressaceae). *American Journal of Botany,* **91**: 1140-1146.

Ecroyd CE. 1982. Biological flora of New Zealand 8. *Agathis australis* (D. Don) Lindl. (Araucariaceae) Kauri. *New Zealand Journal of Botany,* **20**: 17-36.

Edelstein ZR, Ford ED. 2003. Branch and foliage morphological plasticity in old-growth *Thuja plicata*. *Tree Physiology,* **23**: 649-662.

Farjon A. 1989. A second revision of the genus *Keteleeria* Carriere (Taxonomic notes on Pinaceae II*). *Notes from the Royal Botanic Gardens Edinburgh,* **46**: 81-99.

Farjon A. 2010. *A handbook of the world's conifers.* Leiden: Brill.

Fernandes PM, Vega JA, Jiménez E, Rigolot E. 2008. Fire resistance of European pines. *Forest Ecology and Management,* **256**: 246-255.

Frankis MP. 1988. Generic inter-relationships in Pinaceae. *Notes from the Royal Botanic Garden Edinburgh,* **45**: 527-548.

Franklin DA. 1968. Biological flora of New Zealand 3. *Dacrydium cupressinum* Lamb. (Podocarpaceae) Rimu. *New Zealand Journal of Botany,* **6**: 493-513.

Gardner MF, Lara A. 2003. The conifers of Chile: An overview of their distribution and ecology. *Acta Horticulturae (ISHS),* **615**: 165-170.

Gibson N, Brown MJ. 1991. The ecology of *Lagarostrobos franklinii* (Hook.f.) Quinn (Podocarpaceae) in Tasmania. 2. Population structure and spatial pattern. *Australian Journal of Ecology,* **16**: 223-229.

Haase P. 1990. Extreme growth deformations of *Libocedrus bidwillii* at Arthur's Pass, South Island, New Zealand. *New Zealand Journal of Botany,* **28**: 95-98.

Haley C. 1957. *The present status of tree breeding work in Queensland. Paper Seventh British Commonwealth Forestry Conference, Australia and New Zealand.* Australia: Department of Forestry, Brisbane.

Hanson CT, North MP. 2006. Post-fire epicormic branching in Sierra Nevada *Abies concolor* (white fir). *International Journal of Wildland Fire,* **15**: 31-35.

Haverkamp C, Prior LD, Fogliani B, L'Huillier L, Anquez M, Hua Q, Bowman DMJS. 2015. Effect of landscape fires on the demography of the endangered New Caledonian conifer *Callitris sulcata*. *Biological Conservation,* **191**: 130-138.

Hayakawa T, Tomaru N, Yamamoto S. 2004. Stem distribution and clonal structure of *Chamaecyparis pisifera* growing in an old-growth beech-conifer forest. *Ecological Research,* **19**: 411-420.

He LY, Tang CQ, Wu ZL, Wang HC, Ohsawa M, Yan K. 2015. Forest structure and

regeneration of the Tertiary relict *Taiwania cryptomerioides* in the Gaoligong Mountains, Yunnan, southwestern China. *Phytocoenologia,* **45**: 135-155.

He T, Pausas JG, Belcher CM, Schwilk DW, Lamont BB. 2012. Fire-adapted traits of *Pinus* arose in the fiery Cretaceous. *New Phytologist,* **194**: 751-759.

Hill KD. 1997. Architecture of the Wollemi pine (*Wollemia nobilis*, Araucariaceae), a unique combination of model and reiteration. *Australian Journal of Botany,* **45**: 817-826.

Hirayama K, Sakimoto M. 2003. Regeneration of *Cryptomeria japonica* on a sloping topography in a cool-temperate mixed forest in the snowy region of Japan. *Canadian Journal of Forest Research,* **33**: 543-551.

Hitsuma G, Morisawa T, Yagihashi T. 2015. Orthotropic lateral branches contribute to shade tolerance and survival of *Thujopsis dolabrata* var. *hondai* saplings by altering crown architecture and promoting layering. *Botany,* **93**: 353-360.

Holtmeier F-K, Broll G. 2017. Layering in the Rocky Mountain treeline ecotone: clonal conifer groups' distribution, structure, and functional role. *Trees-Structure and Function,* **31**: 953-965.

House S, Dieters M, Johnson M, Haines R. 1998. Inhibition of orthotropic replacement shoots with auxin treatment on decapitated hoop pine, *Araucaria cunninghamii*, for seed orchard management. *New Forests,* **16**: 221-230.

Ishii H, Ford ED. 2001. The role of epicormic shoot production in maintaining foliage in old *Pseudotsuga menziesii* (Douglas-fir) trees. *Canadian Journal of Botany,* **79**: 251-264.

Jagels R, Day ME. 2004. The adaptive physiology of *Metasequoia* to Eocene high-latitude environments. In: Hemsley AR, Poole I, eds. *The evolution of plant physiology*. London: Elsevier.

Jeník J. 1994. Clonal growth in woody plants - a review. *Folia Geobotanica & Phytotaxonomica,* **29**: 291-306.

Keeley JE, Keeley MB, Bond WJ. 1999. Stem demography and post-fire recruitment of a resprouting serotinous conifer. *Journal of Vegetation Science,* **10**: 69-76.

Keppel G. 2001. Notes on the natural history of *Cycas seemannii* (Cycadaceae). *South Pacific Journal of Natural and Applied Sciences,* **19**: 35-41.

Kirkpatrick JB. 1984. Tasmanian high mountain vegetation II - Rocky Hill and Pyramid Mountain. *Papers and Proceedings of the Royal Society of Tasmania,* **118**: 5-20.

Kirkpatrick JB, Bridle KL, Dickinson KJM. 2010. Decades-scale vegetation change in burned and unburned alpine coniferous heath. *Australian Journal of Botany,* **58**: 453-462.

Krapek J, Buma B. 2018. Limited stand expansion by a long-lived conifer at a leading northern range edge, despite available habitat. *Journal of Ecology,* **106**: 911-924.

Kruse S, Kolmogorov AI, Pestryakova LA, Herzschuh U. 2020. Long-lived larch clones may conserve adaptations that could restrict treeline migration in northern Siberia.

Ecology and Evolution, **10**: 10017-10030.

Lacey CJ, Johnston RD. 1990. Woody clumps and clumpwoods. *Australian Journal of Botany,* **38**: 299-334.

Ladd PG, Enright NJ. 2011. Ecology of fire-tolerant podocarps in temperate Australian forests. In: Turner BL, Cernusak LA, eds. *Ecology of the Podocarpaceae in tropical forests*. Washington DC: Smithsonian Institution Scholarly Press.

Lamb FH. 1899. Root suckers on douglas fir. *Botanical Gazette,* **28**: 69-70.

Land WJG. 1913. Vegetative reproduction in an *Ephedra*. *Botanical Gazette,* **55**: 439-445.

Landesmann JB, Gowda JH, Garibaldi LA, Kitzberger T. 2015. Survival, growth and vulnerability to drought in fire refuges: implications for the persistence of a fire-sensitive conifer in northern Patagonia. *Oecologia,* **179**: 1111-1122.

Lanner RM. 1995. The role of epicormic branches in the life history of western larch. In: Schmidt WC, McDonald KJ, eds. *Ecology and Management of Larix forests; a look ahead.*: USDA Forest Service Paper 83.

Lanner RM. 2002. Why do trees live so long? *Ageing Research Reviews,* **1**: 653-671.

Lazzeri-Aerts R, Russell W. 2014. Survival and recovery following wildfire in the southern range of the coast redwood forest. *Fire Ecology,* **10**: 43-55.

Li H-J, Liu P, Zhang Z-X, Chen W-X, Qui Z-J, Liu C-S, Liu J-l, Liao J-P. 2010. Ice and snow damage and subsequent sprouting of *Cunninghamia lanceolata* (Taxodiaceae) plantation and their related-factors analysis. *Acta Botanica Yunnanica,* **32**: 158-166.

Li M, Ritchie GA. 1999. Eight hundred years of clonal forestry in China: I. traditional afforestation with Chinese fir (*Cunninghamia lanceolata* (Lamb.) Hook.). *New Forests,* **18**: 131-142.

Lilly CJ, Will RE, Tauer CG, Guldin JM, Spetich MA. 2012. Factors affecting the sprouting of shortleaf pine rootstock following prescribed fire. *Forest Ecology and Management,* **265**: 13-19.

Lin C, Chan M-H, Chen F-S, Wang Y-N. 2007. Age structure and growth pattern of an endangered species, *Amentotaxus formosana* Li. *Journal of Integrative Plant Biology,* **49**: 157-167.

Luly JG. 2001. On the equivocal fate of late Pleistocene *Callitris* Vent. (Cupressaceae) woodlands in arid South Australia. *Quaternary International,* **83-85**: 155-168.

Lusk CH. 1996. Stand dynamics of the shade-tolerant conifers *Podocarpus nubigena* and *Saxegothaea conspicua* in Chilean temperate rain forest. *Journal of Vegetation Science,* **7**: 549-558.

López-Soria L, Castell C. 1992. Comparative genet survival after fire in woody Mediterranean species. *Oecologia,* **91**: 493-499.

Marin ET. 1961. Vegetative propagation of Norfolk Island pine (*Araucaria excelsa* R. Br.).

Phillipine Journal of Forestry, **17**: 179-189.

Marler TE, Lawrence JH. 2013. Phytophagous insects reduce cycad resistance to tropical cyclone winds and impair storm recovery. *HortScience,* **48**: 1224-1226.

Martin TJ, Ogden J. 2006. Wind damage and response in New Zealand forests: a review. *New Zealand Journal of Ecology,* **30**: 295-310.

McCoy S, Jaffré T, Rigault F, Ash JE. 1999. Fire and succession in the ultramafic maquis of New Caledonia. *Journal of Biogeography,* **26**: 579-594.

McGlone MS, Richardson SJ, Burge OR, Perry GLW, Wilmshurst JM. 2017. Palynology and the ecology of the New Zealand conifers. *Frontiers in Earth Science,* **5**: 94.

McLean JD. 1950. *Age studies in the rhizome of Ephedra coryi.,* Master of Science, Texas University College, Lubbock, Texas.

Midgley JJ, Bond WJ, Geldenhuys CJ. 1995. The ecology of southern African conifers. In: Enright NJ, Hill RS, eds. *Ecology of the southern conifers.* Melbourne: Melbourne University Press.

Mill RR, Thomas P. 1999. 370. *Falcatifolium taxoides* Podocarpaceae. *Curtis's Botanical Magazine,* **16**: 199-211.

Minore D, Weatherly HG. 1996. Stump sprouting of Pacific yew. *USDA Forest Service Pacific Northwest Research Station General Technical Report PNW-GTR-378.*

Molloy BPJ. 1995. *Manoao* (Podocarpaceae), a new monotypic conifer genus endemic to New Zealand. *New Zealand Journal of Botany,* **33**: 183-201.

Molloy BPJ. 1996. A new species name in *Phyllocladus* (Phyllocladaceae) from New Zealand. *New Zealand Journal of Botany,* **34**: 287-297.

Momose Y. 1978. Vegetative propagation of Malaysian trees. *Malaysian Forester,* **41**: 219-223.

Ne'eman G, Fotheringham CJ, Keeley JE. 1999. Patch to landscape patterns in post fire recruitment of a serotinous conifer. *Plant Ecology,* **145**: 235-242.

Nikles DG. 1961. *The development of a new method for grafting hoop and kauri pines. Research Note 10.* Brisbane: Queensland Forest Service.

Norton DA, Herbert JW, Beveridge AE. 1988. The ecology of *Dacrydium cupressinum*: a review. *New Zealand Journal of Botany,* **26**: 37-62.

Nowakowska M, Baran J. 2007. Frost damage of trees and shrubs grown at the Dendrological Garden in Glinna during the winter 2005/2006. *Rocznik Dendrologiczny,* **55**: 129-140.

Ogden J, Fordham RA, Pilkington S, Serra RG. 1991. Forest gap formation and closure along an altitudinal gradient in Tongariro National Park, New Zealand. *Journal of Vegetation Science,* **2**: 165-172.

O'Hara KL, Cox LE, Nikolaeva S, Bauer JJ, Hedges R. 2017. Regeneration dynamics of coast redwood, a sprouting conifer species: A review with implications for management and restoration. *Forests,* **8**: 144.

O'Hara KL, Valappil NI. 2000. Epicormic sprouting of pruned western larch. *Canadian Journal of Forest Research,* **30**: 324-328.

Page CN. 1990. Phyllocladaceae. In: Kramer KU, Green PS, eds. *The families and genera of vascular plants. Pteridophytes and gymnosperms. Volume 1.* Berlin: Springer-Verlag.

Parker T, Donoso C. 1993. Natural regeneration of *Fitzroya cupressoides* in Chile and Argentina. *Forest Ecology and Management,* **59**: 63-85.

Parmenter RR. 2008. Long-term effects of a summer fire on desert grassland plant demographics in New Mexico. *Rangeland Ecology & Management,* **61**: 156-168.

Paula S, Naulin PI, Arce C, Galaz C, Pausas JG. 2016. Lignotubers in Mediterranean basin plants. *Plant Ecology,* **217**: 661-676.

Pausas JC, Llovet J, Rodrigo A, Vallejo R. 2008. Are wildfires a disaster in the Mediterranean basin? - A review. *International Journal of Wildland Fire,* **17**: 713-723.

Pausas JG, Su WH, Luo C, Shen Z. 2021. A shrubby resprouting pine with serotinous cones endemic to south-west China. *Ecology,* **102**: e03282.

Perry GLW, Wilmshurst JM, McGlone MS. 2014. Ecology and long-term history of fire in New Zealand. *New Zealand Journal of Ecology,* **38**: 157-176.

Peterson CJ, Pickett STA. 1991. Treefall and resprouting following catastrophic windthrow in an old-growth hemlock-hardwoods forest. *Forest Ecology and Management,* **42**: 205-217.

Phillips FJ. 1911. Two sprouting conifers of the southwest. *Botanical Gazette,* **51**: 385-390.

Piirto DD, Hawksworth WJ, Hawksworth MM. 1986. Giant sequoia sprouts. Does thinning trigger stump sprouting? *Journal of Forestry,* **84**: 24-25.

Potzger JE. 1937. Vegetative reproduction in conifers. *American Midland Naturalist,* **18**: 1001-1004.

Prior LD, Bowman DMJS. 2020. Classification of post-fire responses of woody plants to include pyrophobic communities. *Fire,* **3**: 15.

Prior LD, Bowman DMJS, Brook BW. 2007. Growth and survival of two north Australian relictual tree species, *Allosyncarpia ternata* (Myrtaceae) and *Callitris intratropica* (Cupressaceae). *Ecological Research,* **22**: 228-236.

Quine CP. 2004. Development of epicormic sprouts on Sitka spruce stems in response to windthrown gap formation. *Forestry,* **77**: 225-233.

Randall CK, Duryea ML, Vince SW, English RJ. 2005. Factors influencing stump sprouting by pondcypress (*Taxodium distichum* var. *nutans* (Ait.) Sweet). *New*

Forests, **29**: 245-260.

Read J, Hill RS. 1988. The dynamics of some rainforest associations in Tasmania. *Journal of Ecology,* **76**: 558-584.

Ritland C, Pape T, Ritland K. 2001. Genetic structure of yellow cedar (*Chamaecyparis nootkatensis*). *Canadian Journal of Botany,* **79**: 822-828.

Rodríguez-Trejo DA, Pausas JG, Miranda-Moreno AG. 2019. Plant responses to fire in a Mexican arid shrubland. *Fire Ecology,* **15**: 11.

Rourke JP. 1991. *Tetraclinis articulata*, a hitherto unrecorded naturalized alien conifer in South Africa. *Bothalia,* **21**: 62-64.

Rundel PW. 2019. A Neogene heritage: conifer distributions and endemism in Mediterranean-climate ecosystems. *Frontiers in Ecology and Evolution,* **7**: 364.

Sagheb-Talebi K, Amirghasemi F, Dargahi D. 2001. Investigation on the structure of young stands in the mountainous forest of Arasbaran (Northwest Iran). *Schweizerische Zeitschrift für Forstwesen,* **152**: 383-388.

Savill P. 2015. *Cryptomeria japonica* (Thunb. ex L.f.) D.Don Japanese red cedar, or sugi. *Quarterly Journal of Forestry,* **109**: 97-102.

Schubert TH, Zambrana JA. 1982. *Araucaria heterophylla* and *Pinus caribaea*: potential Christmas trees for Puerto Rico. *Journal of Agriculture of the University of Puerto Rico,* **66**: 145-149.

Schwartz MW, Hermann SM, Van Mantgem PJ. 2000. Population persistence in Florida torreya: Comparing modeled projections of a declining coniferous tree. *Conservation Biology,* **14**: 1023-1033.

Shapcott A. 1991. Dispersal and establishment of Huon pine (*Lagarostrobos franklinii*). *Papers and Proceedings of the Royal Society of Tasmania,* **125**: 17-26.

Shiembo PN. 1999. The sustainability of eru (*Gnetum africanum* and *Gnetum buchholzianum*): over-exploited non-wood forest product from the forests of Central Africa. In: Sunderland TCH, Clark LE, Vantomme P, eds. *Non-wood forest products of Central Africa. Current research issues and prospects for conservation and development.* Rome: FAO.

Silla F, Fraver S, Lara A, Allnutt TR, Newton A. 2002. Regeneration and stand dynamics of *Fitzroya cupressoides* (Cupressaceae) forests of southern Chile's Central Depression. *Forest Ecology and Management,* **165**: 213-224.

Sirois L. 1997. Distribution and dynamics of balsam fir (*Abies balsamea* [L.] Mill.) at its northern limit in the James Bay area. *Ecoscience,* **4**: 340-352.

Smith MA, Wright HA, Schuster JL. 1975. Reproductive characteristics of redberry juniper. *Journal of Range Management,* **28**: 126-128.

Soto DP, Le Quesne C, Lara A, Gardner MF. 2007. Precarious conservation status of *Pilgerodendron uviferum* forests in their northern distribution in the Chilean Coastal

Range. *Bosque,* **28**: 263-270.

Stanek W. 1968. Development of black spruce layers in Quebec and Ontario. *Forestry Chronicle,* **44**: 25-28.

Stevenson DW. 2020. Observations on vegetative branching in cycads. *International Journal of Plant Sciences,* **181**: 564-580.

Stone EL, Stone MH. 1954. Root collar sprouts in pine. *Journal of Forestry,* **52**: 487-491.

Taira H, Tsumura Y, Tomaru N, Ohba K. 1997. Regeneration system and genetic diversity of *Cryptomeria japonica* growing at different altitudes. *Canadian Journal of Forest Research,* **27**: 447-452.

Tang CQ, Yang Y, Ohsawa M, Momohara A, Yi S, Robertson K, Song K, Zhang S, He L. 2015. Community structure and survival of Tertiary relict *Thuja sutchuenensis* (Cupressaceae) in the subtropical Daba Mountains, southwestern China. *Plos One,* **10**: e0125307.

Tang CQ, Yang YC, Momohara A, Wang HC, Luu HT, Li SF, Song K, Qian SH, LePage B, Dong YF, Han PB, Ohsawa M, Le BT, Tran HD, Dang MT, Peng MC, Wang CY. 2019. Forest characteristics and population structure of *Glyptostrobus pensilis*, a globally endangered relict species of southeastern China. *Plant Diversity,* **41**: 237-249.

Teixeira AMC, Curran TJ, Jameson PE, Meurk CD, Norton DA. 2020. Post-fire resprouting in New Zealand woody vegetation: Implications for restoration. *Forests,* **11**: 269.

Temiño-Villota S, Rodríguez-Trejo DA, Molina-Terrén DM, Ryan KC. 2016. Modelling initial mortality of *Abies religiosa* in a crown fire in Mexico. *Forest Systems,* **25**: e047.

Thomas PA, Polwart A. 2003. *Taxus baccata* L. *Journal of Ecology,* **91**: 489-524.

Thomson LAJ. 2006. *Agathis macrophylla* (Pacific kauri). In: Elevitch CR, ed. *Traditional trees of Pacific islands. Their culture, environment and use.* Hawai'i: Permanent Agricultural Resources.

Tolsma A, Coates F, Sutter G. 2004. Recovery of mountain plum-pine shrubland after wildfire (Cobberas). *Arthur Rylah Institute for Environmental Research Technical Report Series No. 153*. Heidelberg: Department of Sustainability and Environment.

Tomlinson PB. 2001. Reaction tissues in *Gnetum gnemon* - A preliminary report. *IAWA Journal,* **22**: 401-413.

Tomlinson PB, Huggett BA. 2011. Partial shoot reiteration in *Wollemia nobilis* (Araucariaceae) does not arise from 'axillary meristems'. *Annals of Botany,* **107**: 909-916.

Veblen TT. 1982. Regeneration patterns in *Araucaria araucana* forests in Chile. *Journal of Biogeography,* **9**: 11-28.

Veblen TT, Armesto JJ, Burns BR, Kitzberger T, Lara A, Leon B, Young KR. 2005. The coniferous forests of South America. In: Anderson F, Gessel S, eds. *Ecosytems of the World 6 Coniferous Forests*. Amsterdam: Elsevier.

Veblen TT, Ashton DH. 1982. The regeneration status of *Fitzroya cupressoides* in the Cordillera Pelada, Chile. *Biological Conservation,* **23**: 141-161.

Veblen TT, Schlegel FM, Escobar B. 1980. Structure and dynamics of old-growth *Nothofagus* forests in the Valdivian Andes, Chile. *Journal of Ecology,* **68**: 1-31.

Veillon. J-M. 1978. Architecture of the New Caledonian species of *Araucaria*. In: Tomlinson PB, Zimmerman MH, eds. *Tropical trees as living systems.* Cambridge: Cambridge University Press.

Venator CR. 1977. Formation of root storage organs and sprouts in *Pinus oocarpa* seedlings. *Turrialba,* **27**: 41-45.

Wardle P. 1963. Growth habits of New Zealand subalpine trees and shrubs. *New Zealand Journal of Botany,* **1**: 18-47.

Waring KM, O'Hara KL. 2005. Ten-year growth and epicormic sprouting response of western larch to pruning in western Montana. *Western Journal of Applied Forestry,* **20**: 228-232.

Weatherspoon CP. 1990. *Sequoiadendron giganteum* (Lindl.) Buchholz giant sequoia. In: Burns RM, Honkala BH, eds. *Silvics of North America: I. conifers. Agriculture Handbook 654.* Washington DC: USDA Forest Service.

Wendling I, Brondani GE. 2015. Vegetative rescue and cuttings propagation of *Araucaria angustifolia* (Bertol.) Kuntze. *Revista Árvore,* **39**: 93-104.

Wendling I, Dutra LF, Hoffmann HA, Bettio G, Hansel F. 2009. Indução de brotações epicórmicas ortotrópicas para a propagação vegetativa de árvores adultas de *Araucaria angustifolia*. *Agronomía Costarricense,* **33**: 309-319.

Windels SK, Flaspohler DJ. 2011. The ecology of Canada yew (*Taxus canadensis* Marsh.): A review. *Botany,* **89**: 1-17.

Wong KM. 1994. A note on root sucker production in the conifer *Dacrydium xanthandrum* (Podocarpaceae) on Mount Kinabalu, Sabah. *Sandakania,* **4**: 87-89.

Wood MK, Scanlon R, Cram DS. 2012. Occurrence of sprouts and seedlings of pinyon pines, alligator junipers, and gray oaks following harvest of fuelwood and prescribed burning. *Southwestern Naturalist,* **57**: 51-57.

Worth JRP, Jordan GJ, Marthick JR, Sakaguchi S, Colhoun EA, Williamson GJ, Ito M, Bowman DMJS. 2017. Fire is a major driver of patterns of genetic diversity in two co-occurring Tasmanian palaeoendemic conifers. *Journal of Biogeography,* **44**: 1254-1267.

Worth JRP, Marthick JR, Harrison PA, Sakaguchi S, Jordan GJ. 2021. The palaeoendemic conifer *Pherosphaera hookeriana* (Podocarpaceae) exhibits high genetic diversity despite Quaternary range contraction and post glacial bottlenecking.

Conservation Genetics, **22**: 307-321.

Worth JRP, Marthick JR, Rossetto M, Cohen J, Bourke G, Jordan GJ. 2018. Development of 15 nuclear EST microsatellite markers for the paleoendemic conifer *Pherosphaera hookeriana* (Podocarpaceae). *Applications in Plant Sciences,* **6**: e1160.

Worth JRP, Sakaguchi S, Rann KD, Bowman CJW, Ito M, Jordan GJ, Bowman DMJS. 2016. Gondwanan conifer clones imperilled by bushfire. *Scientific Reports,* **6**: 33930.

Worth JRP, Yokogawa M, Pérez-Figueroa A, Tsumura Y, Tomaru N, Janes JK, Isagi Y. 2014. Conflict in outcomes for conservation based on population genetic diversity and genetic divergence approaches: a case study in the Japanese relictual conifer *Sciadopitys verticillata* (Sciadopityaceae). *Conservation Genetics,* **15**: 1243-1257.

Wu L, Shinzato T, Chen C, Aramoto M. 2008. Sprouting characteristics of a subtropical evergreen broad-leaved forest following clear-cutting in Okinawa, Japan. *New Forests,* **36**: 239-246.

Yamamoto S. 1998. Regeneration ecology of *Chamaecyparis obtusa* and *Chamaecyparis pisifera* (Hinoki and Sawara cypress), Japan. In: Laderman AD, ed. *Coastally Restricted Forests*. Oxford: Oxford University Press.

Yamamoto S, Moriyama Y, Kobayashi M. 1994. Two types of vegetative reproduction of *Chamaecyparis pisifera* (Sieb. et Zucc.) Endl. *Japanese Society of Forest Environment,* **36**: 57-59.

Zedler PH. 1995. Plant life history and dynamic specialization in the chaparral/coastal sage shrub flora in southern California. In: Kalin Arroyo MT, Zedler PH, Fox MD, eds. *Ecology and biogeography of Mediterranean ecosystems in Chile, California, and Australia.* New York: Springer.

Zhang G, Li Q, Hou X. 2018. Structural diversity of naturally regenerating Chinese yew (*Taxus wallichiana* var. *mairei*) populations in ex situ conservation. *Nordic Journal of Botany,* **36**: e01717.

Zimmer HC, Auld TD, Hughes L, Offord CA, Baker PJ. 2015. Fuel flammability and fire responses of juvenile canopy species in a temperate rainforest ecosystem. *International Journal of Wildland Fire,* **24**: 349-360.
