# Supplementary material for: Gymnosperm Resprouting—A Review
Source: Plants (Basel). 2021 Nov 23;10(12):2551. doi: 10.3390/plants10122551 (PMC8705048; doi:10.3390/plants10122551)
Supplement: Supplementary file 1 [file plants-10-02551-s001.zip › Supplementary plants-1387177/Table S3 Pinus.pdf]

Resprouting in *Pinus*

Supplementary table S3. *Pinus* species that have been reported to be capable of vegetative reproduction. In the references 'USDA FEIS' stands for the United States Department of Agriculture Fire Effects Information System (https://www.feis-crs.org/feis/) which was mainly accessed during January-February 2017.

**Species**	**Location**	**Resprouting type**	**References**
P. canariensis	Macaronesia	Resprout – basal and epicormic	Pryor (1940), Keeley and Zedler (1998), Climent et al. (2004), Fernandes et al. (2008), He et al. (2012, 2016), Grivet et al. (2013)
P. caribaea	Cent. Amer.	Grass stage	Klaus (1989)
		Resprout – basal and epicormic	Haines et al. (1993), Keeley and Zedler (1998), Rodríguez-Trejo and Fulé (2003), He et al. (2012, 2016)
P. clausa	SE USA	Resprout	Keeley and Zedler (1998)
		Does not reproduce vegetatively	Burns and Honkala (1990)
P. cubensis	Cuba	Grass stage	Klaus (1989)
P. devoniana *(=michocana)*	Mexico	Grass stage	Keeley and Zedler (1998), Rodríguez-Trejo and Fulé (2003), He et al. (2012), Poulos et al. (2018)
P. echinata	E & SE USA	Resprout – basal (basal crook) and epicormic	Stone and Stone (1954), Little and Somes (1956), Little and Mergen (1966), Keeley and Zedler (1998), Cain and Shelton (2000), He et al. (2012), Lilly et al. (2012)
P. elliottii	SE USA	Grass stage (+/-), basal	Ketcham and Bethune (1963), Keeley and Zedler (1998), He et al. (2012)
P. engelmannii	Mexico	Grass stage	McCune (1988), Rodríguez-Trejo and Fulé (2003), He et al. (2012)
P. halepensis	Mediterranean	Sprouts from branch dwarf shoots	Grivet et al. (2013)
		Grass stage (variable)	Poulos et al. (2018)
		Generally considered fire sensitive/obligate seeder	Fernandes et al. (2008)
		Can not sprout when cut/burnt	Lepart and Debussche (1991)
P. hartwegii	Cent. Amer.	Grass stage and resprout – basal	Rodríguez-Trejo and Fulé (2003), Viveros-Viveros et al.

		and epicormic	(2009), He et al. (2012)
P. heldreichii	S Europe	Grass stage	He et al. (2012)
		Does not resprout, even at juvenile stage	Grivet et al. (2013)
P. leiophylla	SW USA, Mexico	Resprout – basal and epicormic	Stone and Stone (1954), Keeley and Zedler (1998), Rodríguez-Trejo and Fulé (2003), Baumgartner and Fulé (2007), He et al. (2012), Jimenez-Casas and Zwiazek (2014), Barton and Poulos (2018)
P. massoniana	C & SE China	Resprout	He et al. (2012, 2016), Lin et al. (2020)
P. merkusii	Malesia, Philippines	Grass stage and resprout	Goldammer and Peñafiel (1990), Koskela et al. (1995), Keeley and Zedler (1998), He et al. (2012)
P. montezumae	Mexico	Grass stage and resprout	Keeley and Zedler (1998), Rodríguez-Trejo and Fulé (2003), Viveros-Viveros et al. (2007), He et al. (2012)
P. mugo	Europe	Layering	Dai et al. (2017)
P. occidentalis	Hispaniola	Resprout	He et al. (2012, 2016)
		Does not resprout, after fire or storms	Kennedy and Horn (2008), Gannon and Martin (2014)
P. oocarpa	C America, Mexico	Resprout – basal and epicormic	Venator (1977), Houkal and Ponce (1985), Keeley and Zedler (1998), Rodríguez-Trejo and Fulé (2003), He et al. (2012), Rodríguez-Trejo et al. (2019)
P. palustris	SE USA	Grass stage	Stone and Stone (1954), Farrar (1975), Keeley and Zedler (1998), He et al. (2012), Knapp et al. (2018), Jin et al. (2019)
P. patula	Mexico	Grass stage and resprout	Rodríguez-Trejo and Fulé (2003), He et al. (2012, 2016), Pausas and Keeley (2017)
P. ponderosa	W N America	Young shoots can resprout after damage but it is generally considered that the species does not reproduce vegetatively	Cooperrider (1938), USDA FEIS
P. pringlei	Mexico	Resprout	Rodríguez-Trejo and Fulé (2003), He et al. (2012)
P. pseudostrobus	Mexico	Recover from crown scorch	Rodríguez-Trejo and Fulé (2003)
		Resprout - basal and epicormic	Poulos et al. (2018)

P. pungens	E USA	Resprout – basal (basal crook)	Stone and Stone (1954), Zobel (1969), Keeley and Zedler (1998)
P. rigida	E N America	Resprout – basal (basal crook) and epicormic	Stone and Stone (1954), Little and Somes (1956), Little and Mergen (1966), Ledig and Little (1979), Keeley and Zedler (1998), He et al. (2012, 2016)
P. roxburghii	Himalaya	Grass stage, resprout	Stone and Stone (1954), Goldammer and Peñafiel (1990), He et al. (2012, 2016), Grivet et al. (2013)
P. serotina	SE USA	Resprout – basal and epicormic	Stone and Stone (1954), Bramlett (1990), Keeley and Zedler (1998), He et al. (2012, 2016)
P. taeda	SE USA	Resprouting of decapitated seedlings, older trees do not resprout	Little and Somes (1960)
P. teocote	Mexico	Resprout – basal and epicormic	Stone and Stone (1954), Rodríguez-Trejo and Fulé (2003), Bravo et al. (2012), He et al. (2012)
P. tropicalis	Caribbean	Grass stage and protected buds	Klaus (1989), Liston et al. (1999), He et al. (2012)
P. virginiana	E USA	Weak resprouter	Stone and Stone (1954), Snow (1960), Keeley and Zedler (1998), He et al. (2012, 2016)
P. yunnanensis	SW China	Grass stage and resprouting	He et al. (2012, 2016), Tang et al. (2013), Pausas et al. (2021)

Grivet et al. (2013) also includes *P. brutia*, *P. pinaster* and *P. pinea* as sprouting from dwarf shoots

Del Tredici (2001) also includes *P. banksiana*, *P. resinosa* and *P. strobus* as seedling resprouters from the collar (junction on the seedling axis between the root and shoot)

He et al. (2016) also includes *P. cembroides, P. coulteri, P. georginae, P. maximartinezii*, *P. praetermissa* and *P. quadrifolia* as epicormic resprouters

References

Barton AM, Poulos HM. 2018. Pine vs. oaks revisited: Conversion of Madrean pine-oak forest to oak shrubland after high-severity wildfire in the Sky Islands of Arizona. *Forest Ecology and Management,* **414**: 28-40.

Baumgartner KH, Fulé PZ. 2007. Survival and sprouting responses of Chihuahua pine after the Rodeo-Chediski fire on the Mogollon Rim, Arizona. *Western North American Naturalist,* **67**: 51-56.

Bramlett DL. 1990. *Pinus serotina* Michx. Pond pine. In: Burns RM, Honkala BH, eds. *Silvics of North America. Volume 1: Conifers. Agriculture Handbook 654 Forest Service United States Department of Agriculture*.

Bravo JEJ, Rodríguez-Trejo DA, Myers RL. 2012. Fire tolerance of three tree species in pine-oak forests of Chignahuapan, Puebla, Mexico. *International Journal of Wildland Fire,* **21**: 873-881.

Burns RM, Honkala BH. 1990. *Silvics of North America. Volume 1: Conifers.*: United States Department of Agriculture Forest Service. Agriculture Handbook 654.

Cain MD, Shelton MG. 2000. Survival and growth of *Pinus echinata* and *Quercus* seedlings in response to simulated summer and winter prescribed burns. *Canadian Journal of Forest Research,* **30**: 1830-1836.

Climent J, Tapias R, Pardos JA, Gil L. 2004. Fire adaptations in the Canary Islands pine (*Pinus canariensis*). *Plant Ecology,* **171**: 185-196.

Cooperrider CK. 1938. Recovery processes of ponderosa pine reproduction following injury to young annual growth. *Plant Physiology,* **13**: 5-27.

Dai L, Palombo C, van Gils H, Rossiter DG, Tognetti R, Luo G. 2017. *Pinus mugo* krummholz dynamics during concomitant change in pastoralism and climate in the Central Apennines. *Mountain Research and Development,* **37**: 75-86.

Del Tredici P. 2001. Sprouting in temperate trees: A morphological and ecological review. *Botanical Review,* **67**: 121-140.

Farrar RM. 1975. Sprouting ability of longleaf pine. *Forest Science,* **21**: 189-190.

Fernandes PM, Vega JA, Jiménez E, Rigolot E. 2008. Fire resistance of European pines. *Forest Ecology and Management,* **256**: 246-255.

Gannon BM, Martin PH. 2014. Reconstructing hurricane disturbance in a tropical montane forest landscape in the Cordillera Central, Dominican Republic: implications for vegetation patterns and dynamics. *Arctic, Antarctic, and Alpine Research,* **46**: 767-776.

Goldammer JG, Peñafiel SR. 1990. Fire in the pine-grassland biomes of tropical and subtropical Asia. In: Goldammer JG, ed. *Fire in the tropical biota. Ecosystem processes and global challenges*. Berlin: Springer-Verlag.

Grivet D, Climent J, Zabal-Aguirre M, Neale DB, Vendramin GG, González-Martínez SC.

2013. Adaptive evolution of Mediterranean pines. *Molecular Phylogenetics and Evolution,* **68**: 555-566.

Haines RJ, Walker SM, Copley TR. 1993. Morphology and rooting of shoots developing in response to decapitation and pruning of Caribbean pine. *New Forests,* **7**: 133-141.

He T, Pausas JG, Belcher CM, Schwilk DW, Lamont BB. 2012. Fire-adapted traits of *Pinus* arose in the fiery Cretaceous. *New Phytologist,* **194**: 751-759.

He TH, Belcher CM, Lamont BB, Lim SL. 2016. A 350-million-year legacy of fire adaptation among conifers. *Journal of Ecology,* **104**: 352-363.

Houkal D, Ponce E. 1985. Basal sprouting in *Pinus oocarpa*. *Turrialba,* **35**: 96-101.

Jimenez-Casas M, Zwiazek JJ. 2014. Adventitious sprouting of *Pinus leiophylla* in response to salt stress. *Annals of Forest Science,* **71**: 811-819.

Jin S, Moule B, Yu D, Wang GG. 2019. Fire survival of longleaf pine (*Pinus palustris*) grass stage seedlings: The role of seedling size, root collar position, and resprouting. *Forests,* **10**: 1070.

Keeley JE, Zedler PH. 1998. Evolution of life histories in *Pinus*. In: Richardson DM, ed. *Ecology and biogeography of Pinus.* Cambridge: Cambridge University Press.

Kennedy LM, Horn SP. 2008. Postfire vegetation recovery in highland pine forests of the Dominican Republic. *Biotropica,* **40**: 412-421.

Ketcham DE, Bethune JE. 1963. Fire resistance of South Florida slash pine. *Journal of Forestry,* **61**: 529-530.

Klaus W. 1989. Mediterranean pines and their history. *Plant Systematics and Evolution,* **162**: 133-163.

Knapp BO, Pile LS, Walker JL, Wang GG. 2018. Fire effects on a fire-adapted species: response of grass stage longleaf pine seedlings to experimental burning. *Fire Ecology,* **14**: 2.

Koskela J, Kuusipalo J, Sirikul W. 1995. Natural regeneration dynamics of *Pinus merkusii* in northern Thailand. *Forest Ecology and Management,* **77**: 169-179.

Ledig FT, Little S. 1979. Pitch pine (*Pinus rigida* Mill.): ecology, physiology, and genetics. In: Forman RTT, ed. *Pine barrens: ecosystem and landscape*. New York: Academic Press.

Lepart J, Debussche M. 1991. Invasion processes as related to succession and disturbance. In: Groves RH, Di Castri F, eds. *Biogeography of Mediterranean invasions*. Cambridge: Cambridge University Press.

Lilly CJ, Will RE, Tauer CG, Guldin JM, Spetich MA. 2012. Factors affecting the sprouting of shortleaf pine rootstock following prescribed fire. *Forest Ecology and Management,* **265**: 13-19.

Lin T, Zheng X, Zheng H. 2020. Seasonal variations in leaf and branch trace elements and the influence of a 3-yr 100% rainfall exclusion on *Pinus massoniana* Lamb. *PeerJ,* **8**: 9935.

Liston A, Robinson WA, Piñero D, Alvarez-Buylla ER. 1999. Phylogenetics of *Pinus* (Pinaceae) based on nuclear ribosomal DNA internal transcribed spacer region sequences. *Molecular Phylogenetics and Evolution,* **11**: 95-109.

Little S, Mergen F. 1966. External and internal changes associated with basal-crook formation in pitch and shortleaf pines. *Forest Science,* **12**: 268-275.

Little S, Somes HA. 1956. Buds enable pitch and shortleaf pines to recover from injury. *Station Paper No. 81, Northeastern Forest Experiment Station, Forest Service United States Department of Agriculture*.

Little S, Somes HA. 1960. Sprouting of loblolly pine. *Journal of Forestry,* **58**: 195-197.

McCune B. 1988. Ecological diversity in North American pines. *American Journal of Botany,* **75**: 353-368.

Pausas JG, Keeley JE. 2017. Epicormic resprouting in fire-prone ecosystems. *Trends in Plant Science,* **22**: 1008-1015.

Pausas JG, Su WH, Luo C, Shen Z. 2021. A shrubby resprouting pine with serotinous cones endemic to south-west China. *Ecology,* **102**: e03282.

Poulos HM, Barton AM, Slingsby JA, Bowman DMJS. 2018. Do mixed fire regimes shape plant flammability and post-fire recovery strategies? *Fire,* **1**: 39.

Pryor LD. 1940. The effect of fire on exotic conifers. *Australian Forestry,* **5**: 37-38.

Rodríguez-Trejo DA, Fulé PZ. 2003. Fire ecology of Mexican pines and a fire management proposal. *International Journal of Wildland Fire,* **12**: 23-37.

Rodríguez-Trejo DA, Muñoz PM, Lara PJM. 2019. Fire effects on the trees of a tropical pine forest and a tropical dry forest at Villaflores, Chiapas, Mexico. *Ciencia Florestal,* **29**: 1033-1047.

Snow AG. 1960. *Silvical characteristics of Virginia pine. Station Paper No. 131 Northeastern Forest Experiment Station, Forest Service USDA.*

Stone EL, Stone MH. 1954. Root collar sprouts in pine. *Journal of Forestry,* **52**: 487-491.

Tang CQ, He L-Y, Su W-H, Zhang G-F, Wang H-C, Peng M-C, Wu Z-L, Wang C-Y. 2013. Regeneration, recovery and succession of a *Pinus yunnanensis* community five years after a mega-fire in central Yunnan, China. *Forest Ecology and Management,* **294**: 188-196.

Venator CR. 1977. Formation of root storage organs and sprouts in *Pinus oocarpa* seedlings. *Turrialba,* **27**: 41-45.

Viveros-Viveros H, Sáenz-Romero C, López-Upton J, Vargas-Henández JJ. 2007. Growth and frost damage variation among *Pinus pseudostrobus*, *P. montezumae* and *P. hartwegii* tested in Michoacán, México. *Forest Ecology and Management,* **253**: 81-88.

Viveros-Viveros H, Sáenz-Romero C, Vargas-Hernández JJ, López-Upton J, Ramírez-Valverde G, Santacruz-Varela A. 2009. Altitudinal genetic variation in *Pinus hartwegii*

Lindl. I: Height growth, shoot phenology, and frost damage in seedlings. *Forest Ecology and Management,* **257**: 836-842.

Zobel DB. 1969. Factors affecting distribution of *Pinus pungens*, an Appalachian endemic. *Ecological Monographs,* **39**: 303-333.
